# Supplementary material for: Electrochemical ammonia synthesis via nitrate reduction on Fe single atom catalyst
Source: Nat Commun. 2021 May 17;12:2870. doi: 10.1038/s41467-021-23115-x (PMC8128876; doi:10.1038/s41467-021-23115-x)
Supplement: Supplementary file 1 — Supplementary Information [file 41467_2021_23115_MOESM1_ESM.pdf]

Supplementary Information

Electrochemical ammonia synthesis via nitrate reduction on Fe single atom catalyst

Zhen-Yu Wu[1], Mohammadreza Karamad[2], Xue Yong[3], Qizheng Huang[1], David A. Cullen[4], Peng Zhu[1], Chuan Xia[1], Qunfeng Xiao[5], Mohsen Shakouri[5], Feng-Yang Chen[1], Jung Yoon (Timothy) Kim[1], Yang Xia[1], Kimberly Heck[1], Yongfeng Hu[5], Michael S. Wong[1], Qilin Li[6], Ian Gates[2], Samira Siahrostami[3]*, and Haotian Wang[1,7,8,9]*

[1]Department of Chemical and Biomolecular Engineering, Rice University, Houston, TX 77005, USA

[2]Department of Chemical and Petroleum Engineering, University of Calgary, 2500 University Drive NW, Calgary, Alberta T2N 1N4, Canada

[3]Department of Chemistry, University of Calgary, 2500 University Drive NW, Calgary, Alberta T2N 1N4, Canada

[4]Center for Nanophase Materials Sciences, Oak Ridge National Laboratory, Oak Ridge, TN, USA

[5]Canadian Light Source Inc., University of Saskatchewan, Saskatoon, SK S7N 2V3, Canada

[6]Department of Civil and Environmental Engineering, Rice University, Houston, TX 77005, USA

[7]Department of Materials Science and NanoEngineering, Rice University, Houston, TX 77005, USA

[8]Department of Chemistry, Rice University, Houston, TX 77005, USA

[9]Azrieli Global Scholar, Canadian Institute for Advanced Research (CIFAR), Toronto, 22 Ontario M5G 1M1, Canada

*Correspondence to: htwang@rice.edu or samira.siahrostami@ucalgary.ca

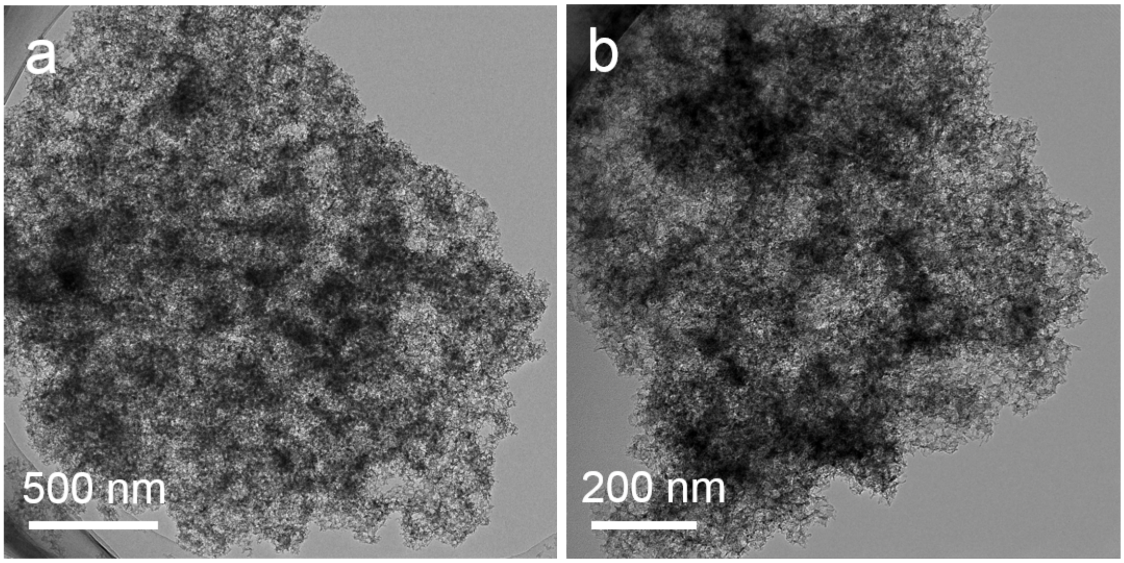

Supplementary Figure 1. (a, b) TEM images of Fe SAC, indicating that it has an interconnected vesicle-like structure.

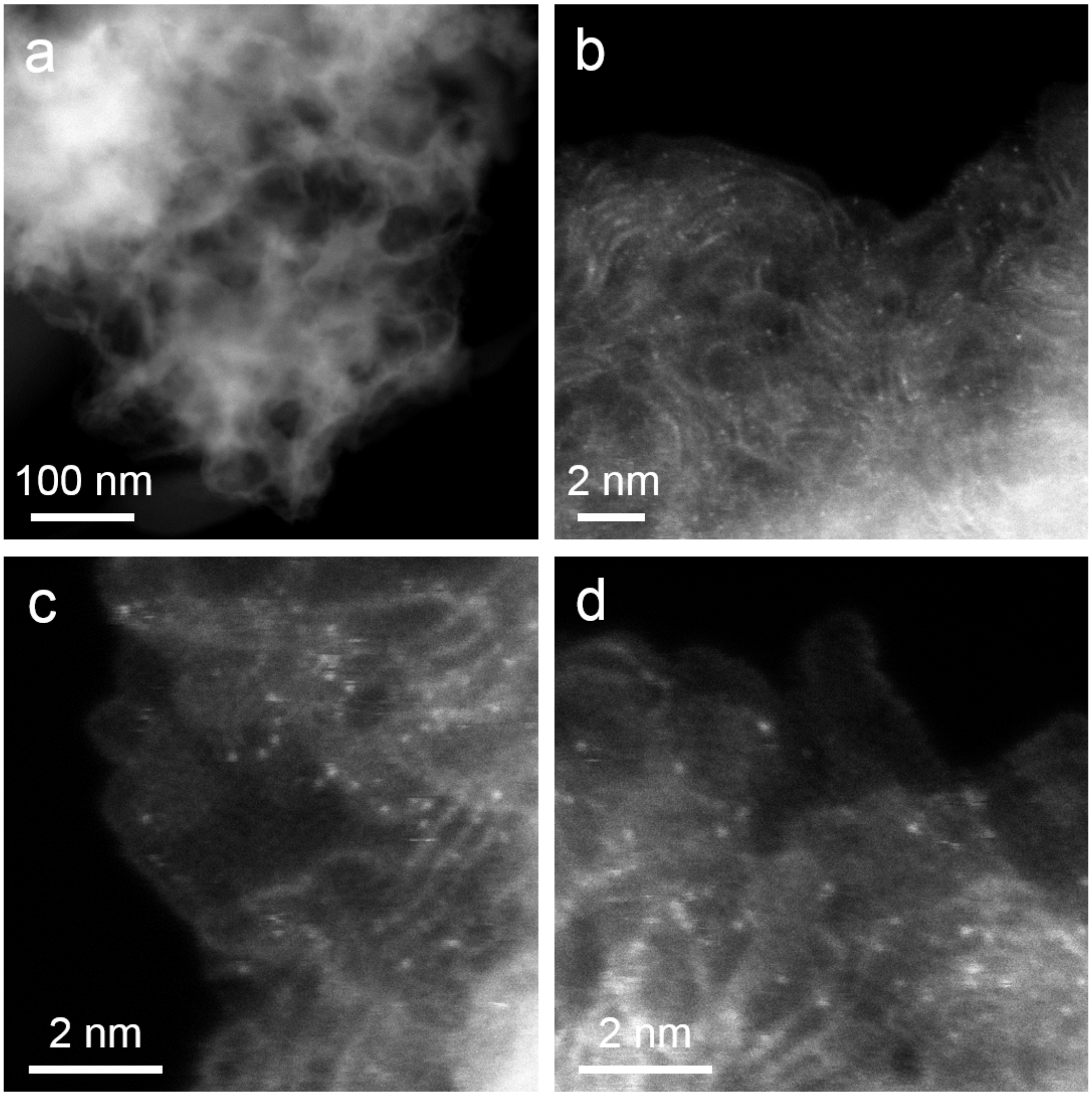

Supplementary Figure 2. (a) MAADF-STEM and (b-d) AC MAADF-STEM images of Fe SAC. We only find individual metal atoms in Fe SAC. No cluster or nanoparticle is observed for Fe SAC.

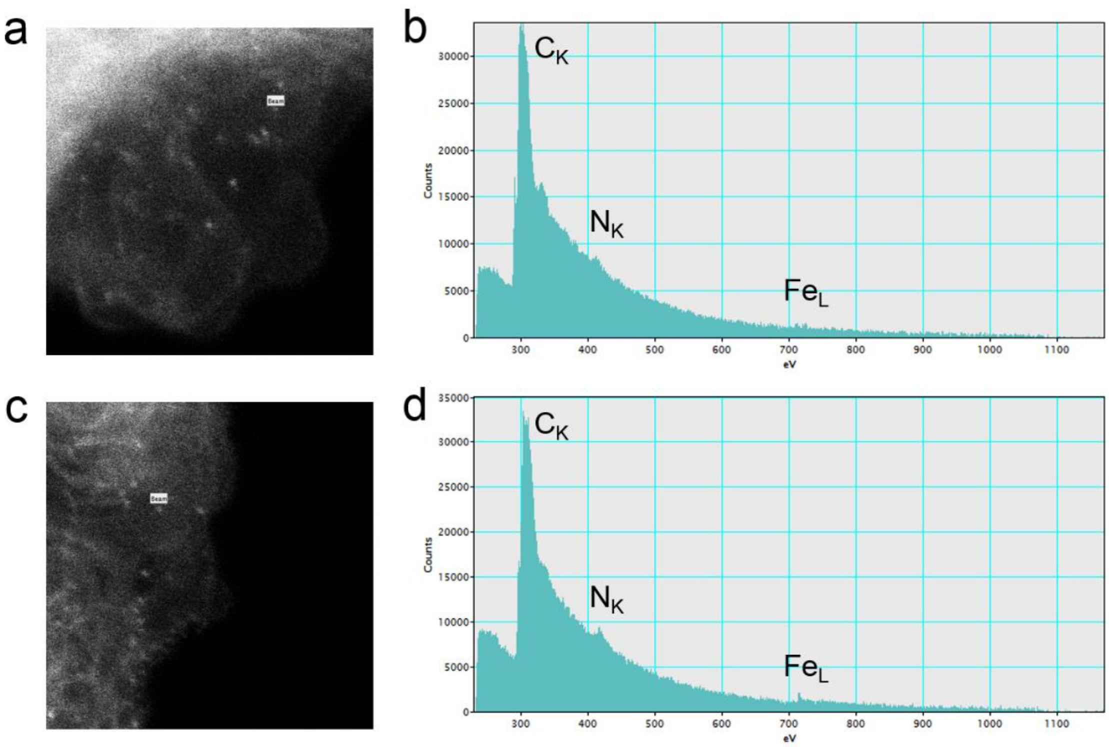

Supplementary Figure 3. (a, c) AC MAADF-STEM images of Fe SAC at different areas. (b, d) EELS point spectra from the marked areas in (a) and (c). Fe-N-C coordinate structures are existed in these areas.

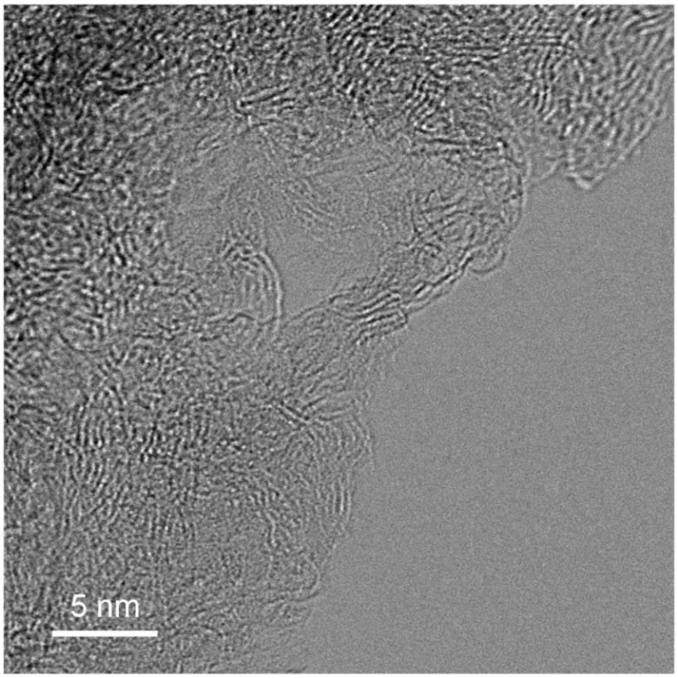

Supplementary Figure 4. HRTEM image of Fe SAC. Randomly orientated graphitic layers are observed in the Fe SAC.

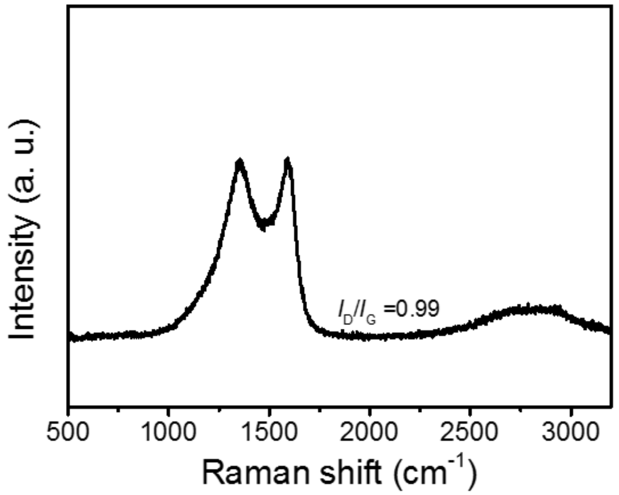

Supplementary Figure 5. Raman spectrum of Fe SAC. The I_D/I_G of Fe SAC is 0.99, indicating its high graphitic degree.

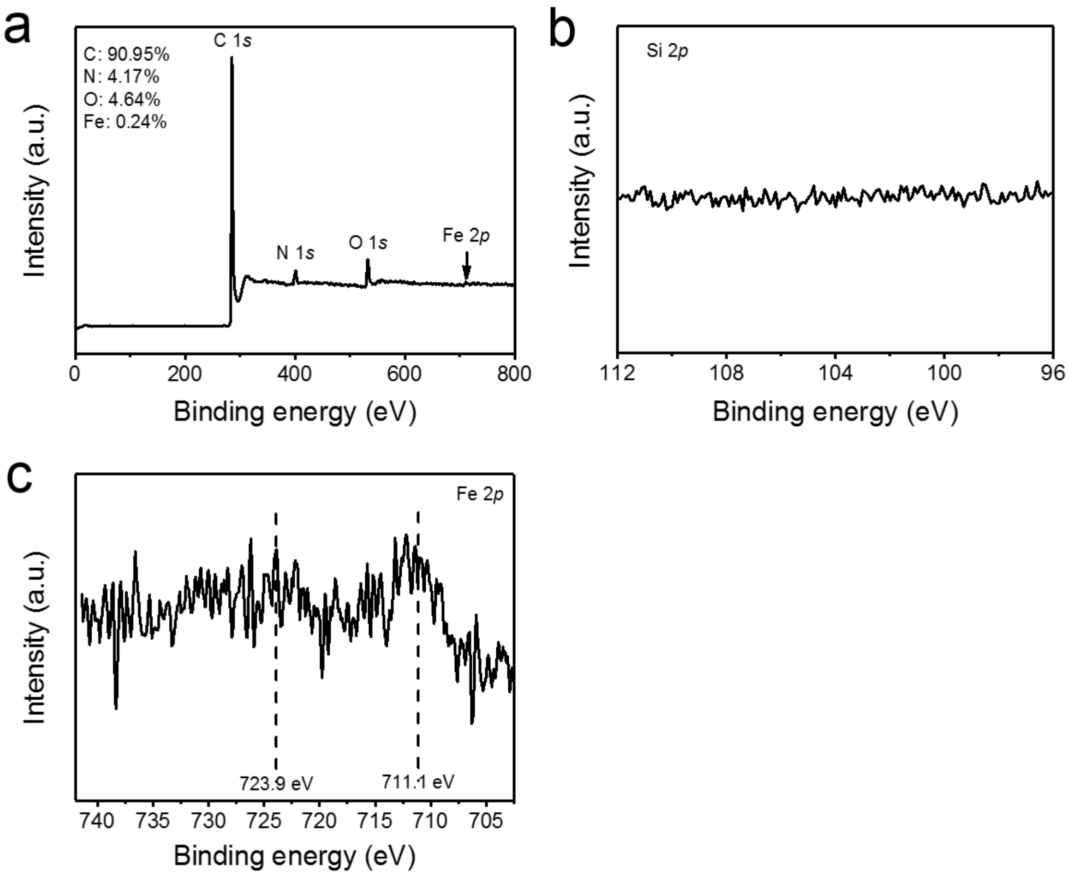

Supplementary Figure 6. (a) XPS survey, (b) Si 2*p*, and (c) Fe 2*p* spectra of Fe SAC.

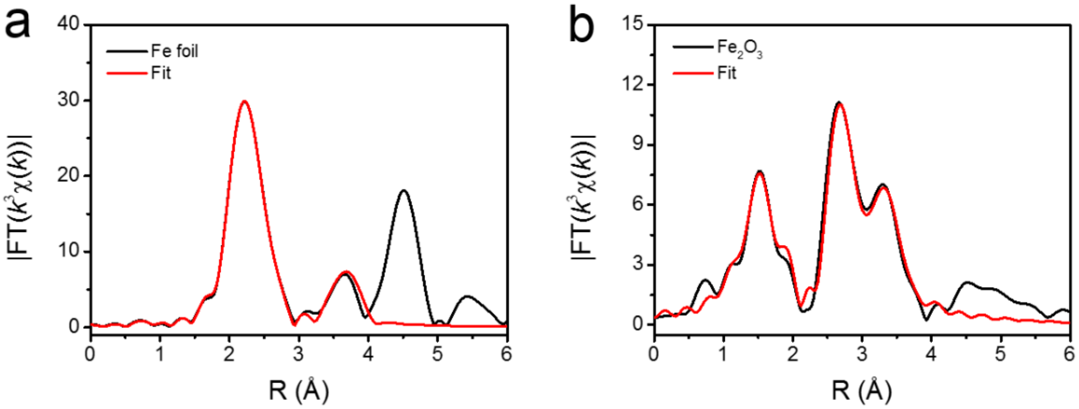

Supplementary Figure 7. Fitting results of the EXAFS spectra of (a) Fe foil and (b) Fe_2O_3.

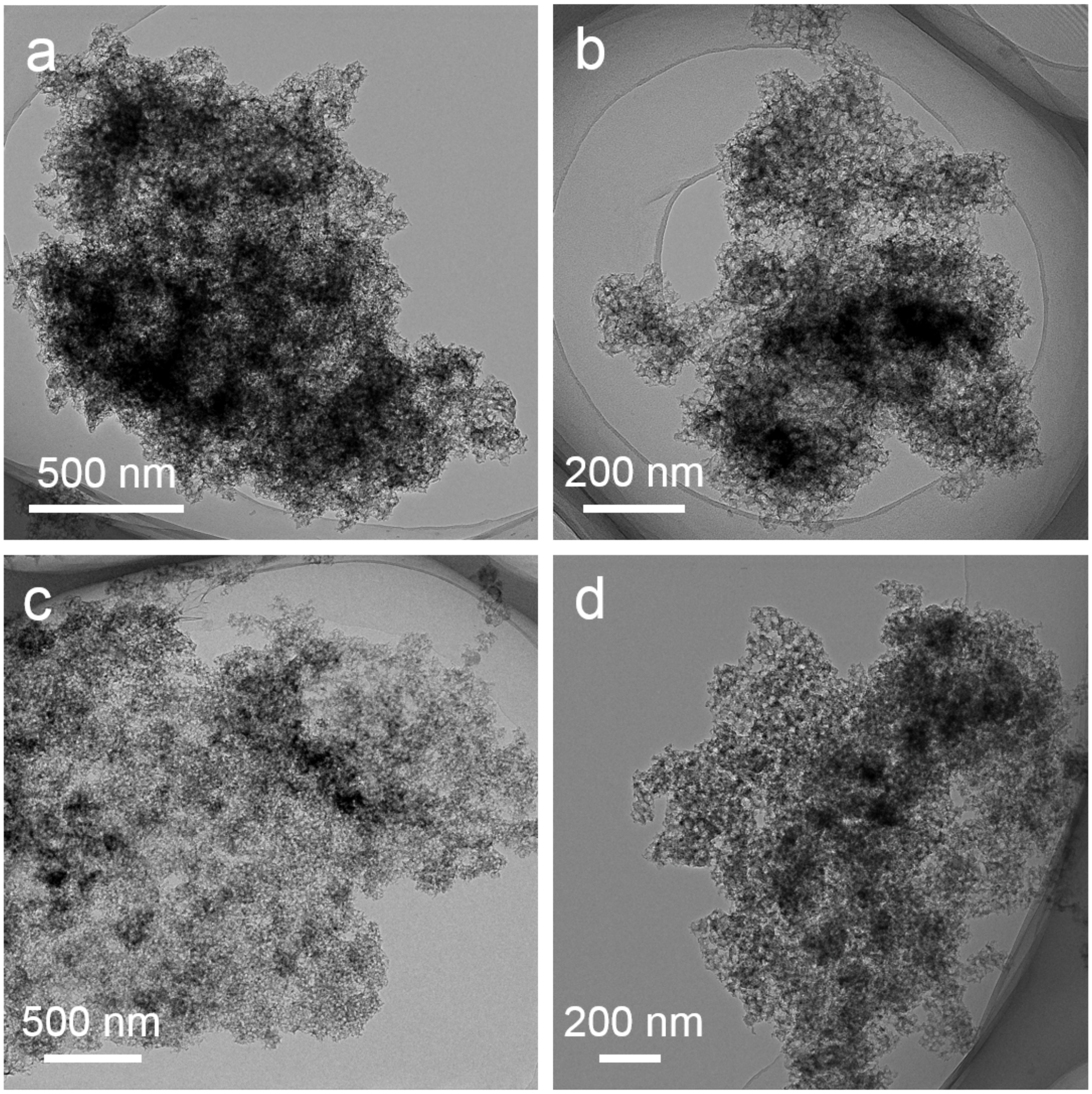

Supplementary Figure 8. TEM images of (a, b) Co SAC and (c, d) Ni SAC, indicating that they have an interconnected vesicle-like structure.

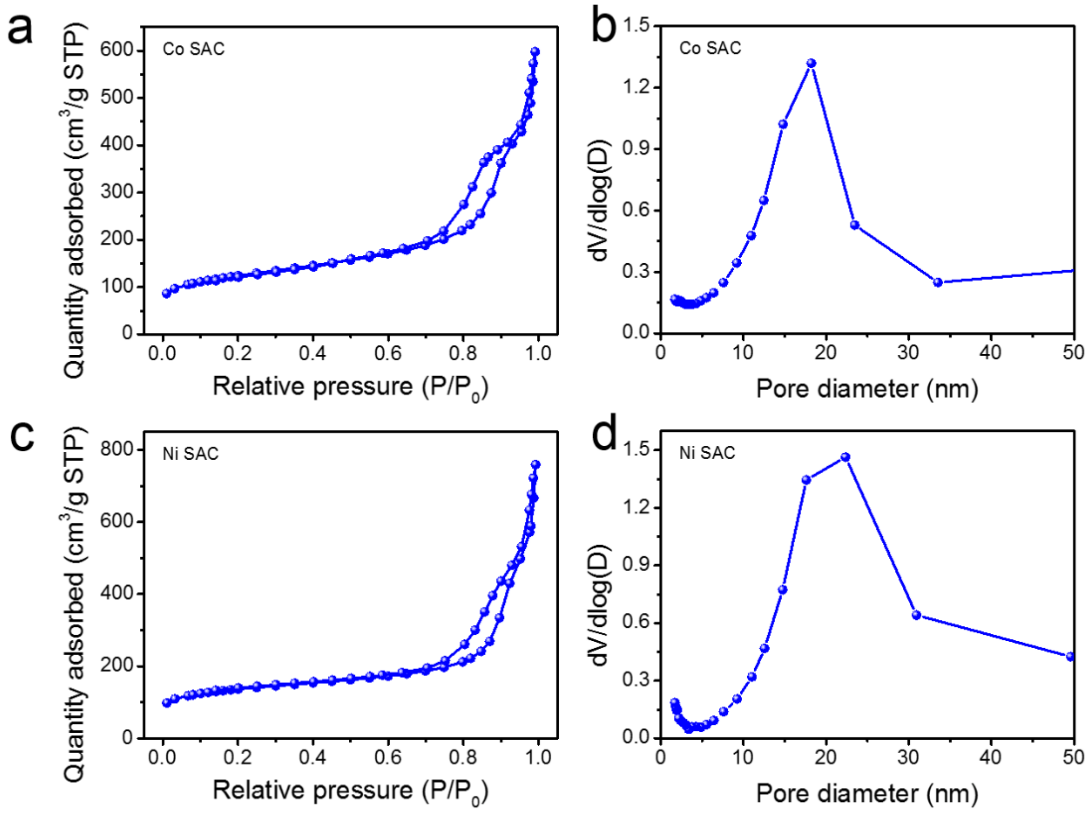

Supplementary Figure 9. N_2 adsorption-desorption isotherms of (a) Co SAC and (c) Ni SAC. Pore-size distribution curves of (b) Co SAC and (d) Ni SAC. The Co SAC and Ni SAC exhibit remarkable hysteresis loops of type-IV, indicating that they possess highly mesoporous structures.

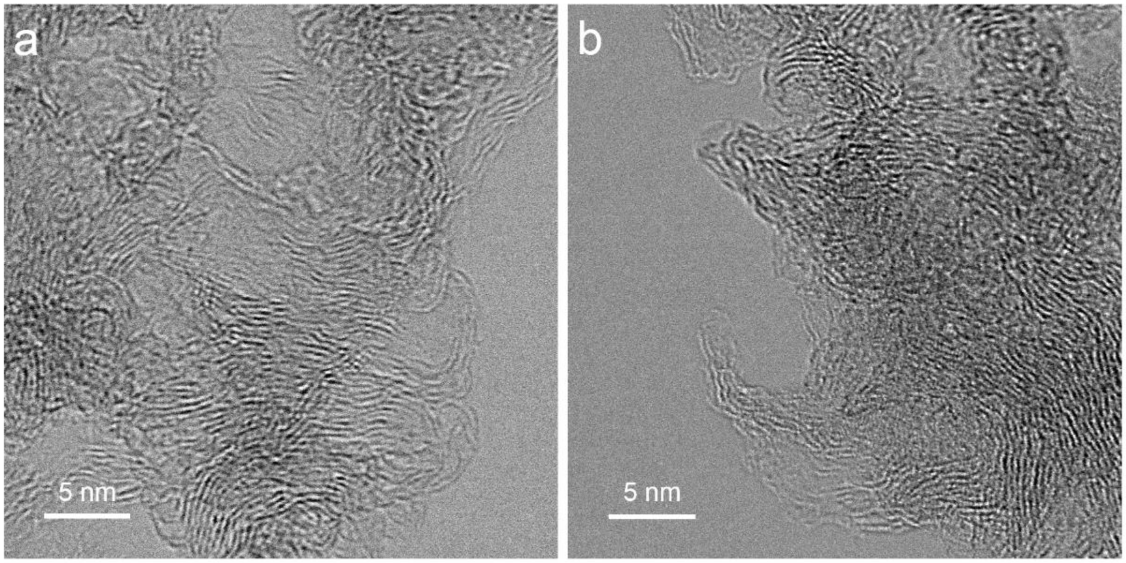

Supplementary Figure 10. HRTEM images of (a) Co SAC and (b) Ni SAC. Randomly orientated graphitic layers are observed in both Co SAC and Ni SAC.

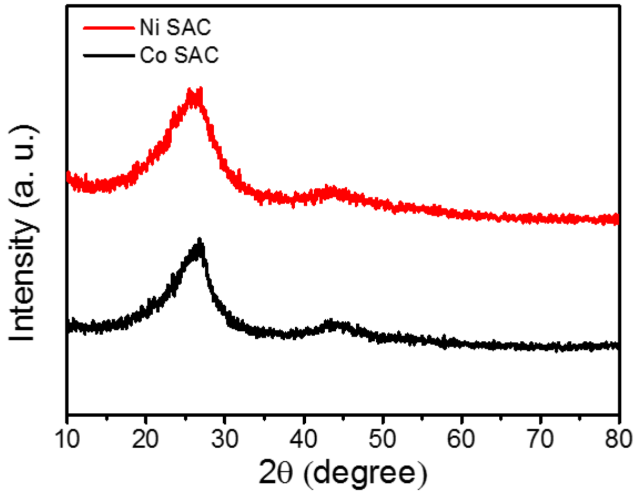

Supplementary Figure 11. XRD patterns of Co SAC and Ni SAC. Two characteristic peaks at ca. 26.2° and 43.7°, attributing to the (002) and (101) planes of graphitic carbon, are observed for Co SAC and Ni SAC. No other peaks of metal-based crystals are found.

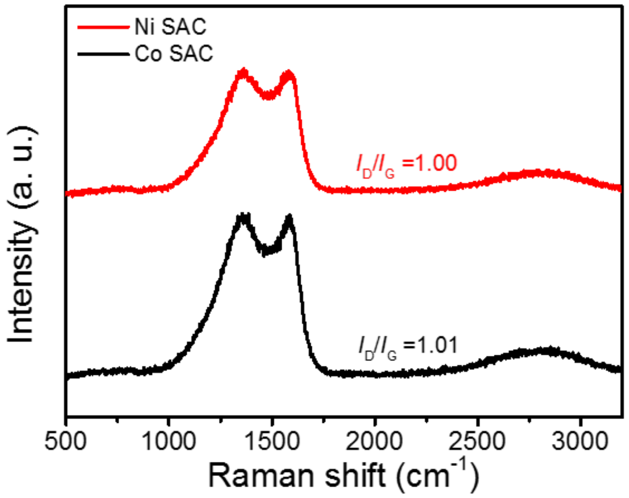

Supplementary Figure 12. Raman spectra of Co SAC and Ni SAC. The I_D/I_G of Co SAC and Ni SAC are 1.01 and 1.00, respectively, indicating their high graphitic degree.

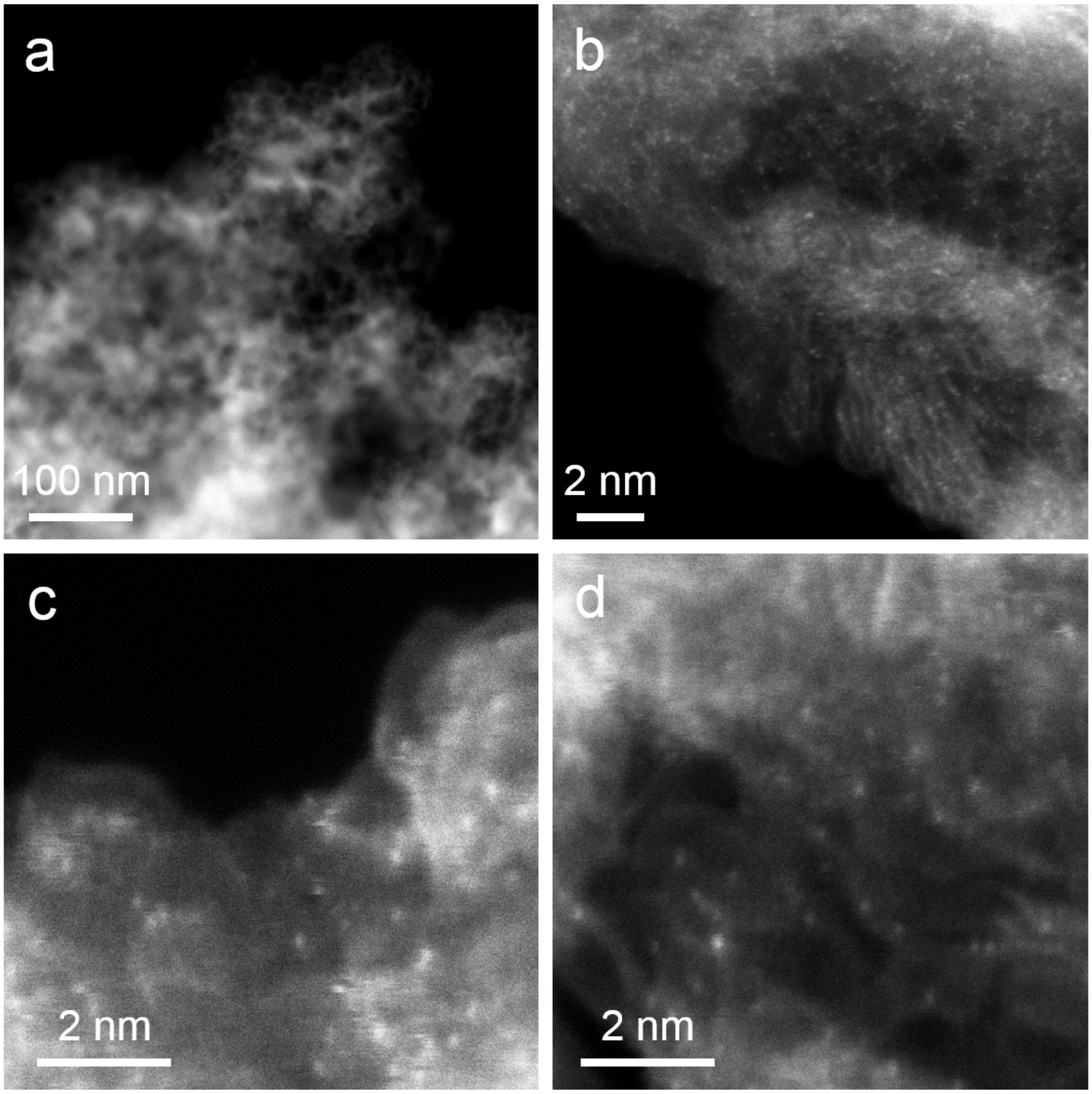

Supplementary Figure 13. (a) MAADF-STEM and (b-d) AC MAADF-STEM images of Co SAC. We only find individual metal atoms in Co SAC. No cluster or nanoparticle is observed for Co SAC.

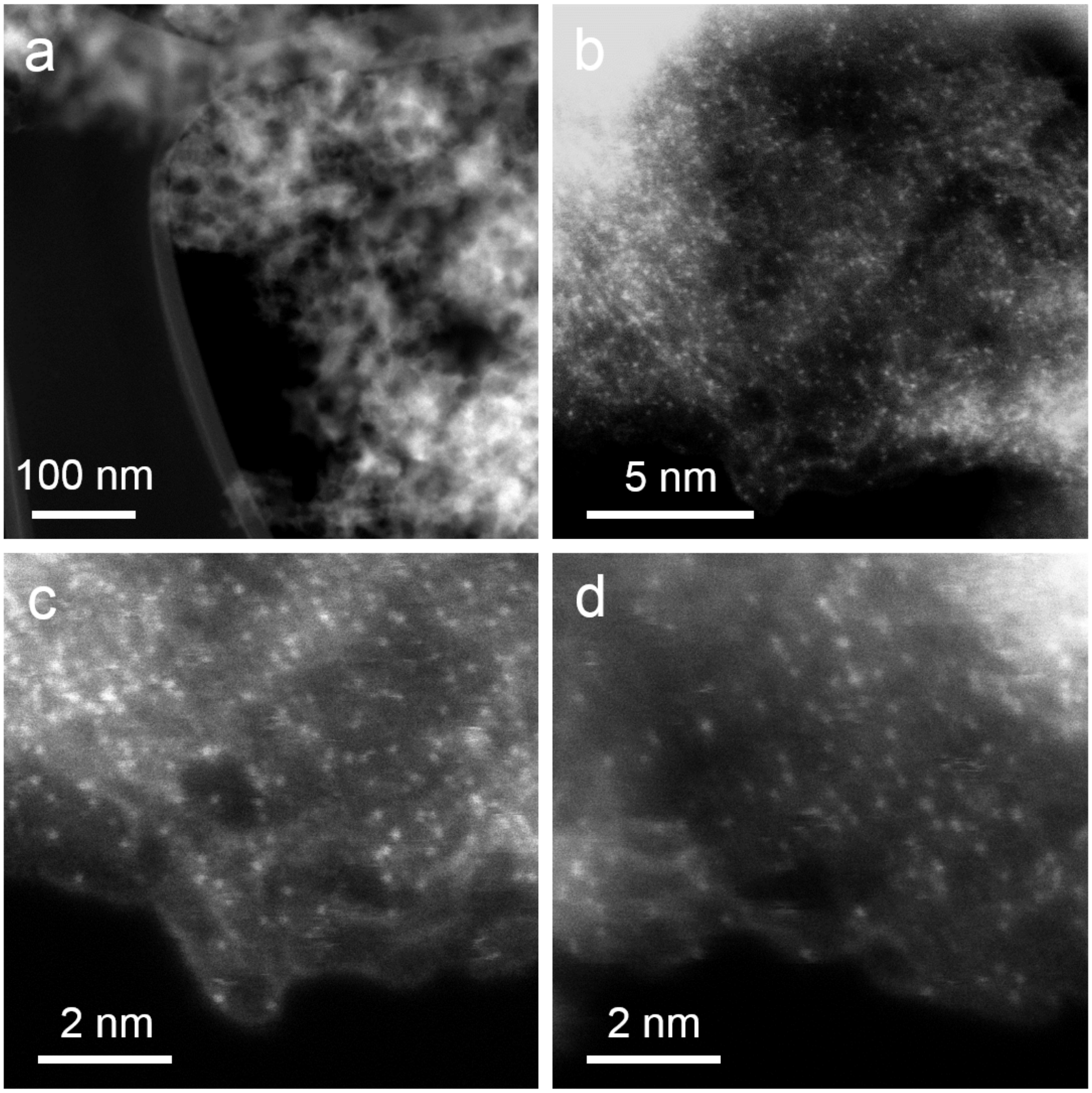

Supplementary Figure 14. (a) MAADF-STEM and (b-d) AC MAADF-STEM images of Ni SAC. We only find individual metal atoms in Ni SAC. No cluster or nanoparticle is observed for Ni SAC.

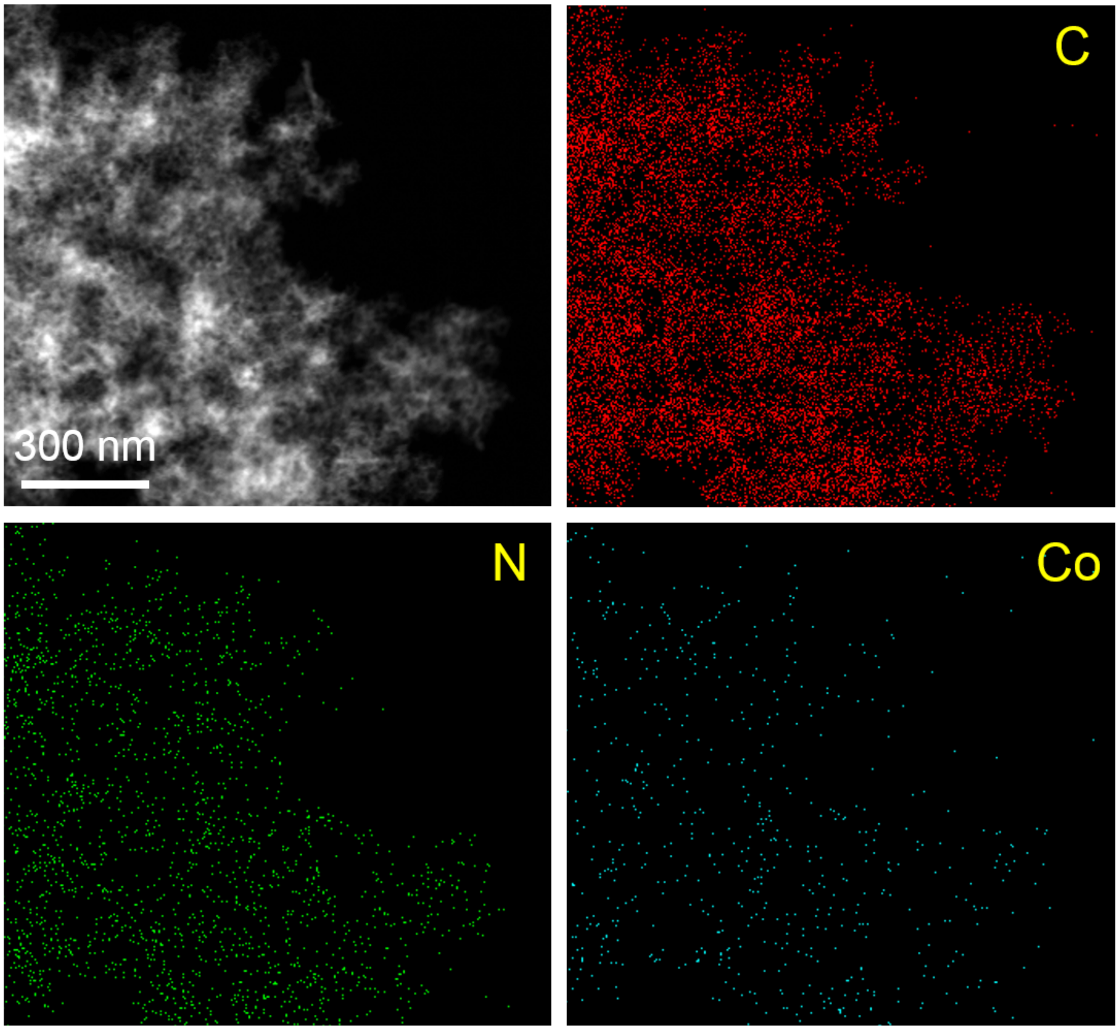

Supplementary Figure 15. EDS mapping images of Co SAC, indicating the existence of Co, N, and C elements throughout the porous structure.

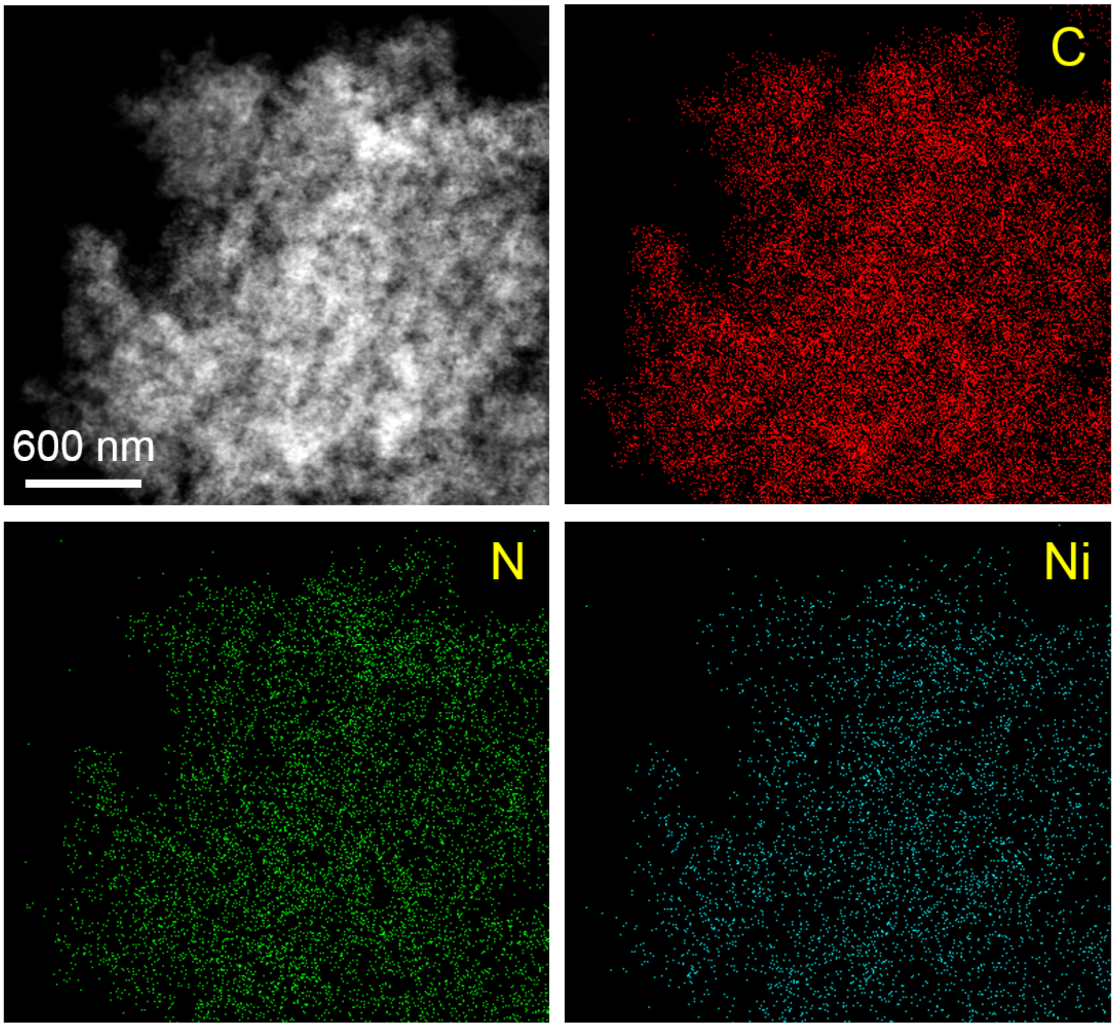

Supplementary Figure 16. EDS mapping images of Ni SAC, indicating the existence of Ni, N, and C elements throughout the porous structure.

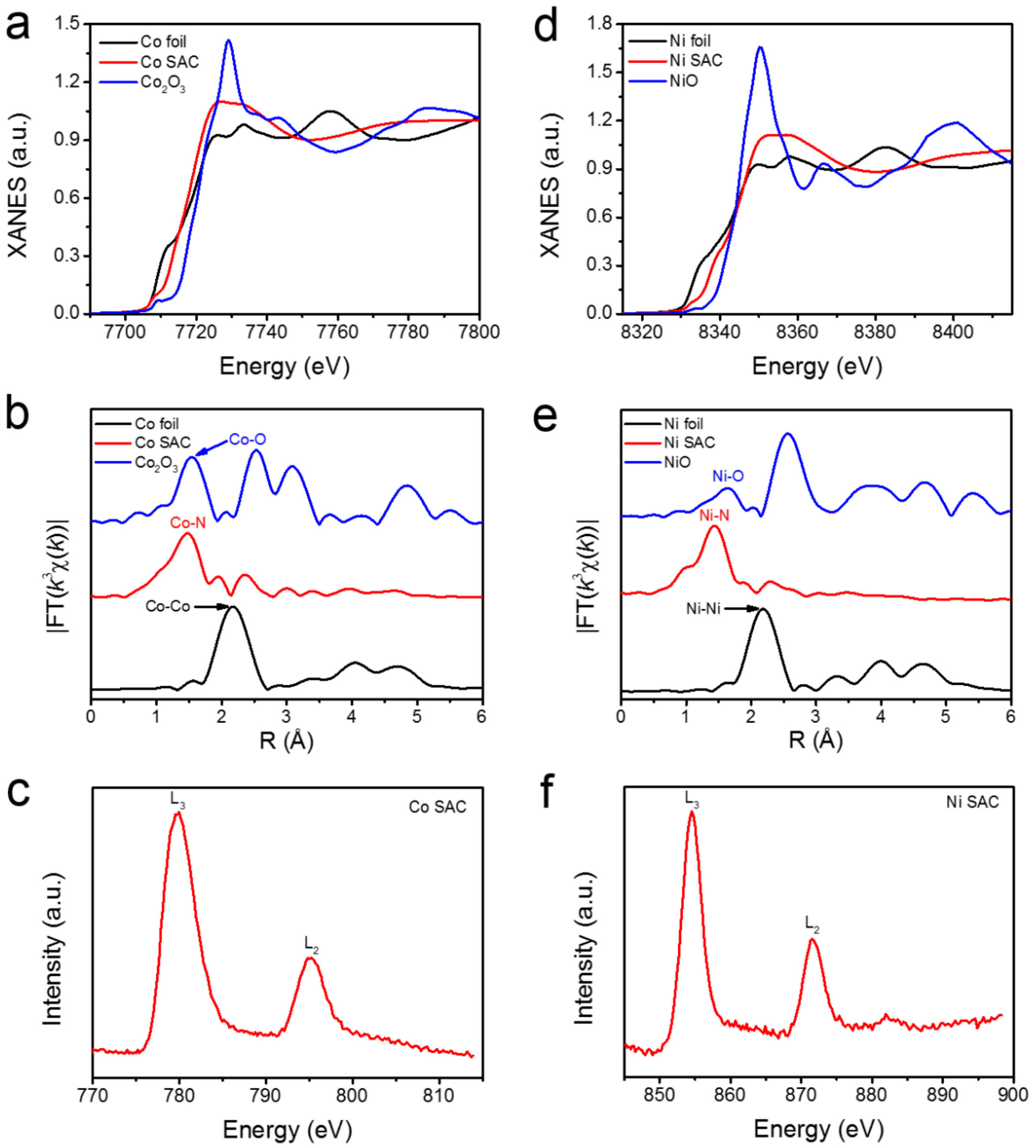

Supplementary Figure 17. (a) XANES spectra at the Co K-edge of the Co SAC, referenced Co foil and Co_2O_3. (b) FT k^3-weighted $\chi(k)$-function of the EXAFS spectra at Co K-edge. (c) XANES spectrum at Co L-edge of Co SAC. (d) XANES spectra at the Ni K-edge of the Ni SAC, referenced Ni foil and NiO. (e) FT k^3-weighted $\chi(k)$-function of the EXAFS spectra at Ni K-edge. (f) XANES spectrum at Ni L-edge of Ni SAC. The above XAFS results demonstrate successful preparation of Co SAC and Ni SAC with exclusive metal-N coordinate structures.

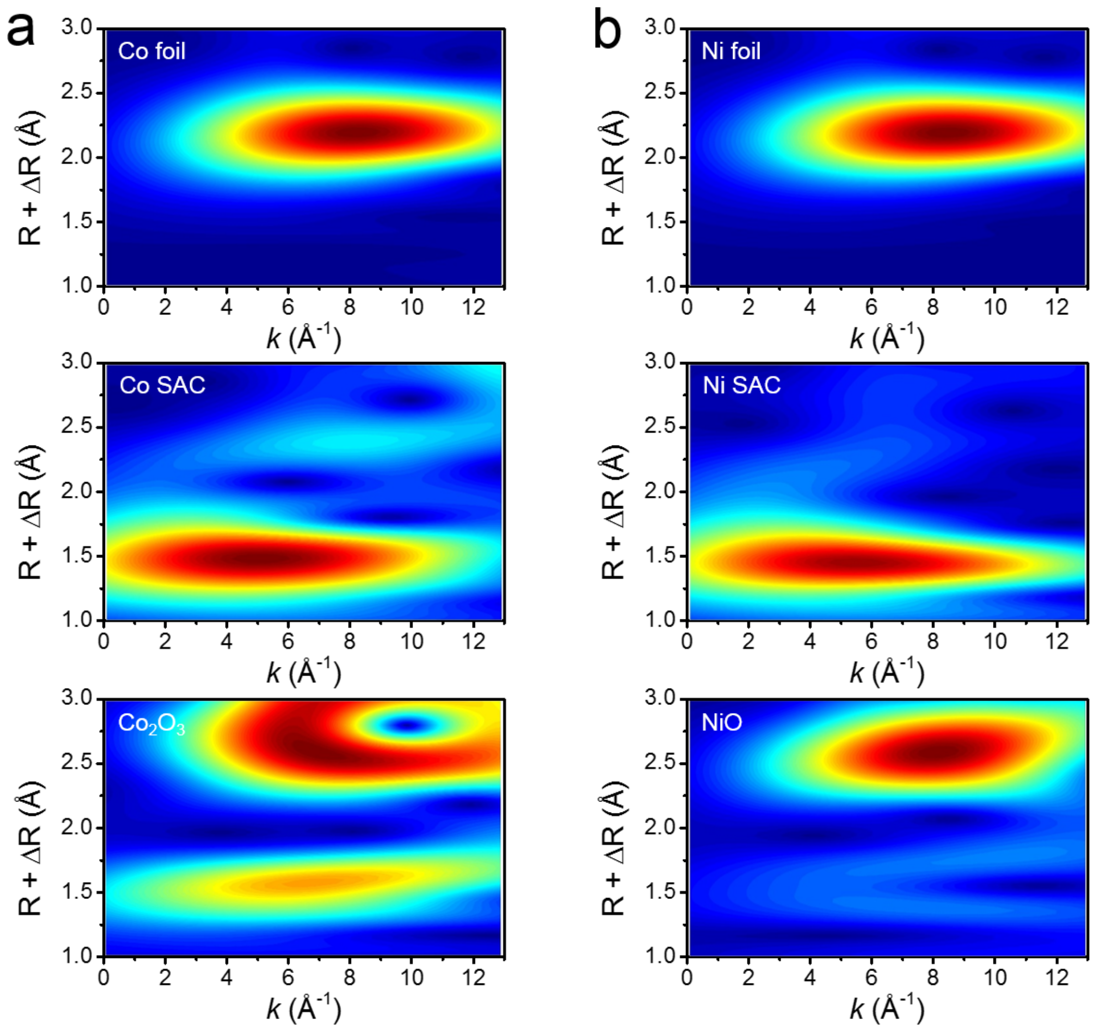

Supplementary Figure 18. (a) WT at the Co K-edge of the Co SAC, referenced Co foil and Co_2O_3. (b) WT at the Ni K-edge of the Ni SAC, referenced Ni foil and NiO. Only metal-N coordination is detected in the WT contour plots of Co SAC and Ni SAC.

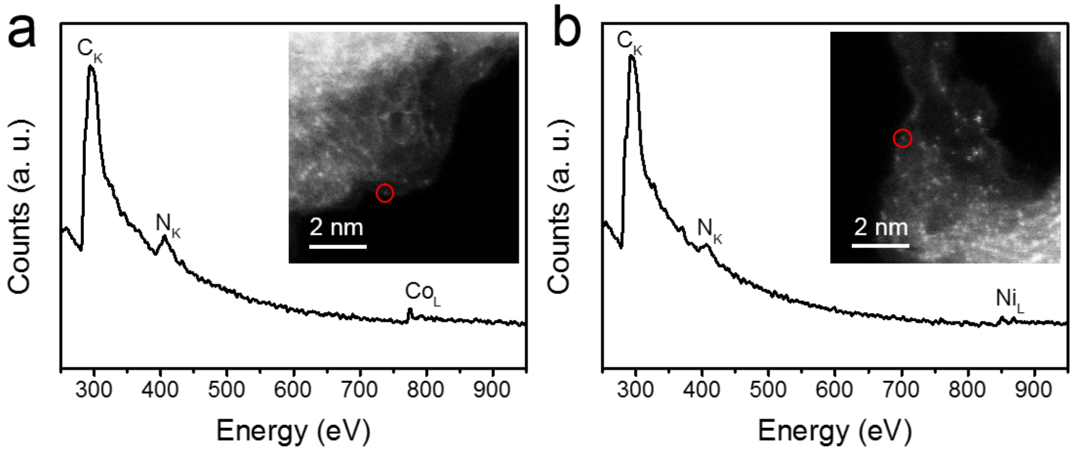

Supplementary Figure 19. EELS point spectra of (a) Co SAC and (b) Ni SAC, demonstrating that the single Co and Ni atoms are coordinated by N atoms.

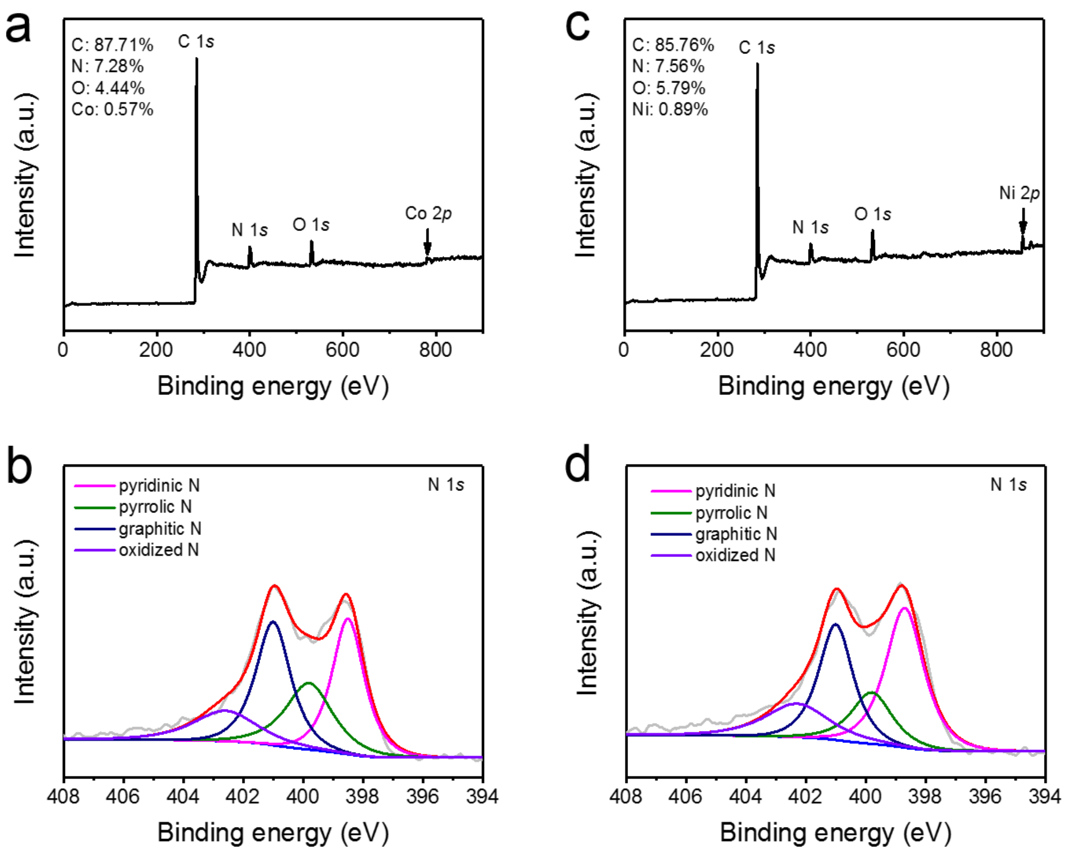

Supplementary Figure 20. (a) XPS survey, (b) high-resolution N 1*s* spectra of Co SAC. (c) XPS survey, (d) high-resolution N 1*s* spectra of Ni SAC.

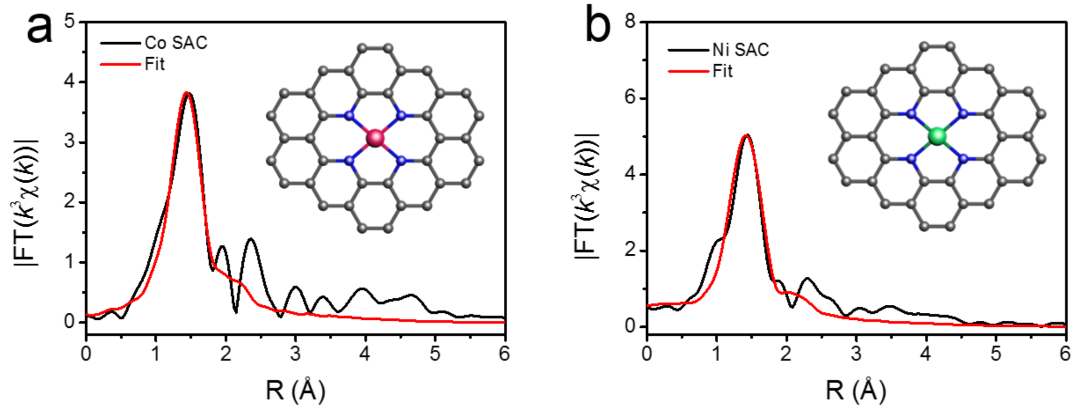

Supplementary Figure 21. Fitting results of the EXAFS spectra of (a) Co SAC and (b) Ni SAC at R space. Insets: Schematic models of Co SAC and Ni SAC: Co (pink), Ni (green), N (blue), and C (gray). According to the fitting results, Co SAC and Ni SAC have Co-N_4 and Ni-N_4 structures, similar to that of Fe SAC.

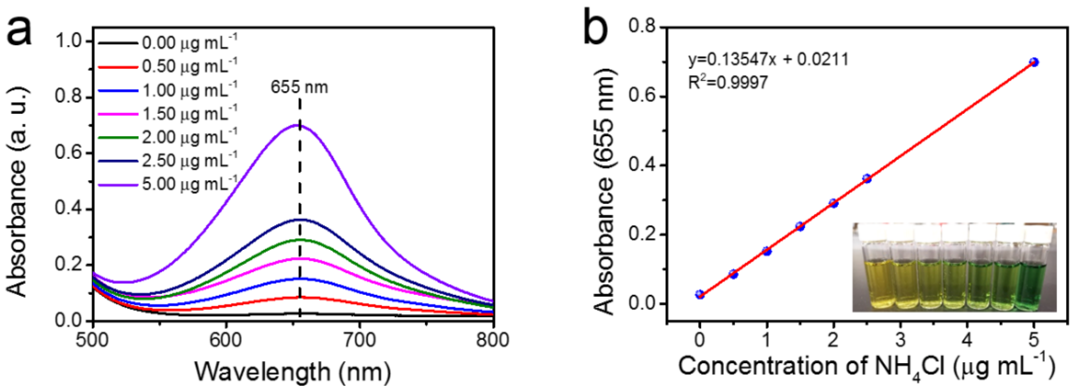

Supplementary Figure 22. UV-vis calibration curve of NH_3 in ultrapure water using ammonium chloride solutions of known concentration as standards. (a) UV-vis curves of indophenol assays with NH_4^+ ions after incubated for 2 hours and (b) calibration curve used for estimation of NH_3 by NH_4^+ ion of different concentrations.

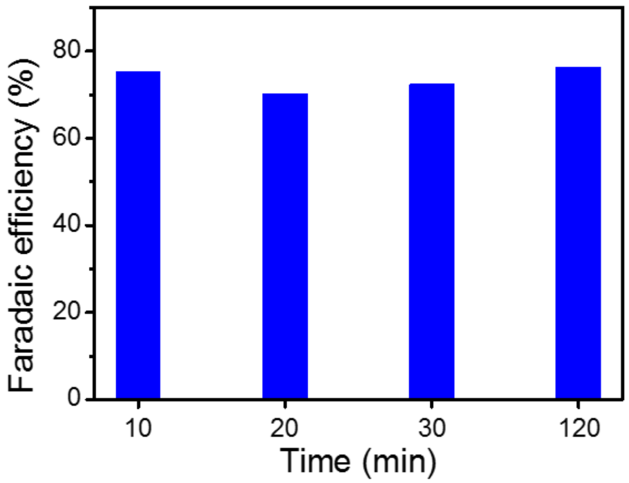

Supplementary Figure 23. NH_3 Faradaic efficiency of NO_3^-RR on Fe SAC at -0.66V at different time.

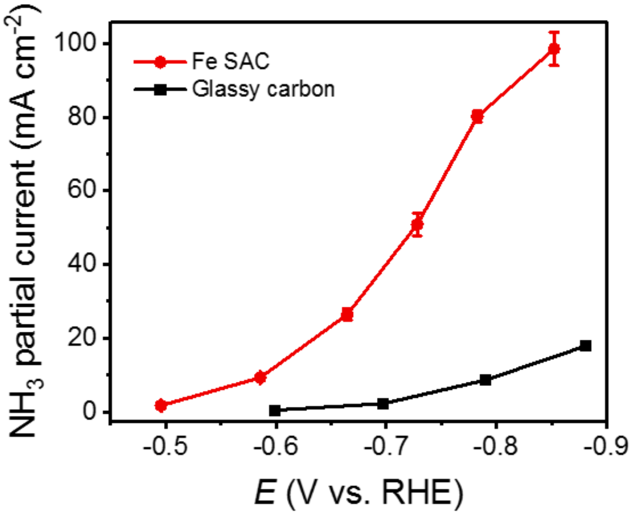

Supplementary Figure 24. Partial current density of Fe SAC and bare glassy carbon (GC) electrode.

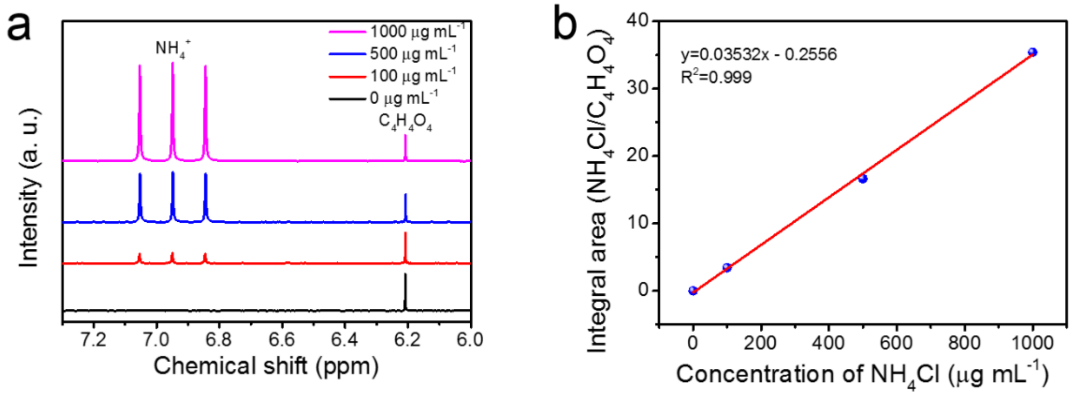

Supplementary Figure 25. NMR calibration curve of NH_3 in 0.5 M KNO_3 solution using ammonium chloride solutions of known concentration as standards. (a) ^1H NMR spectra of NH_4^+ ions using maleic acid as an internal standard and (b) calibration curve used for estimation of NH_3 by NH_4^+ ion of different concentrations.

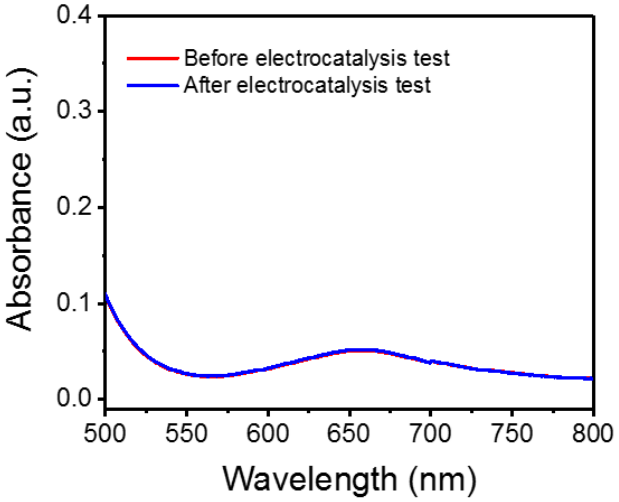

Supplementary Figure 26. UV-vis curves of electrolytes before and after electrocatalysis test for Fe SAC in a K_2SO_4 solution without KNO_3.

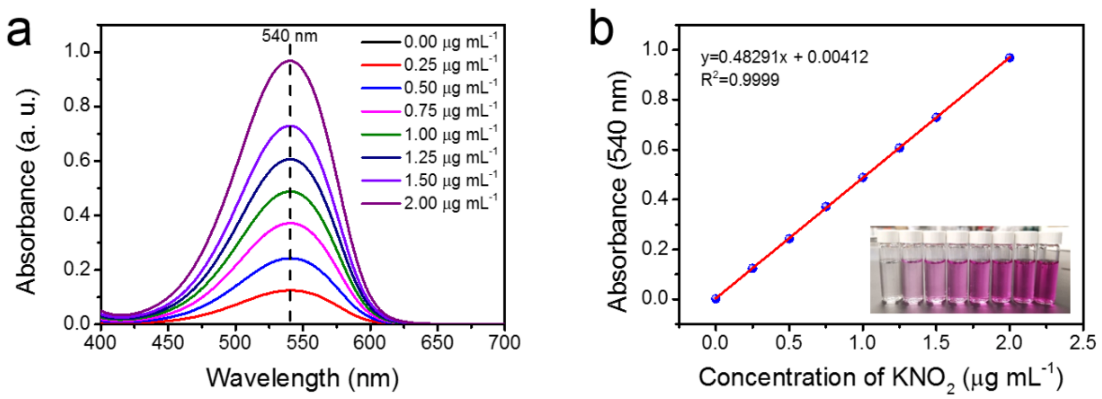

Supplementary Figure 27. UV-vis calibration curve of NO_2^- in ultrapure water using KNO_2 solutions of known concentration as standards. (a) UV-vis curves of NO_2^- ions after sitting for 20 minutes and (b) calibration curve used for estimation of NO_2^- by NO_2^- ion of different concentrations.

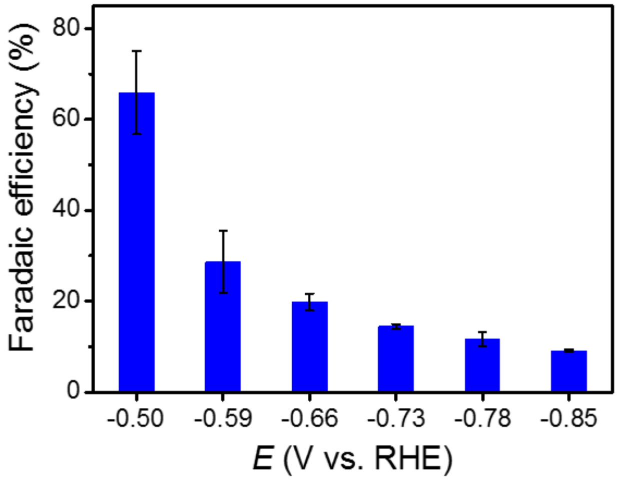

Supplementary Figure 28. NO_2^- FE for nitrate reduction on Fe SAC catalyst at each given potential.

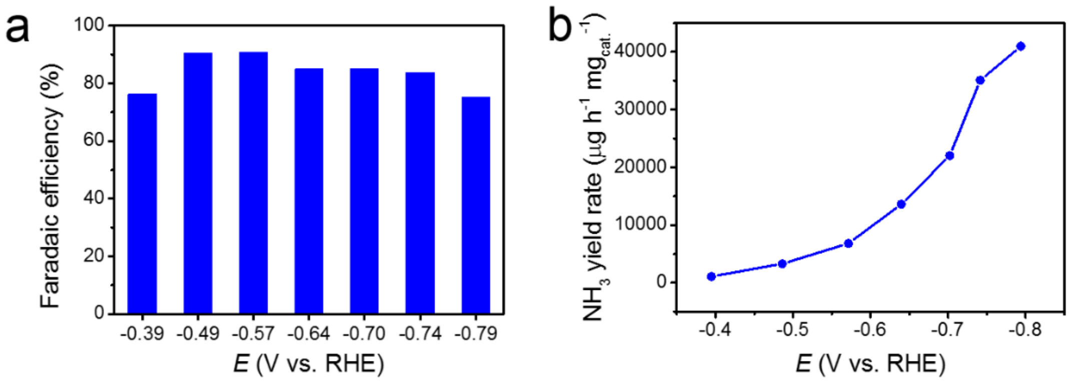

Supplementary Figure 29. NH_3 (a) FE and (b) yield rate of NO_2^- reduction on Fe SAC catalyst at each given potential.

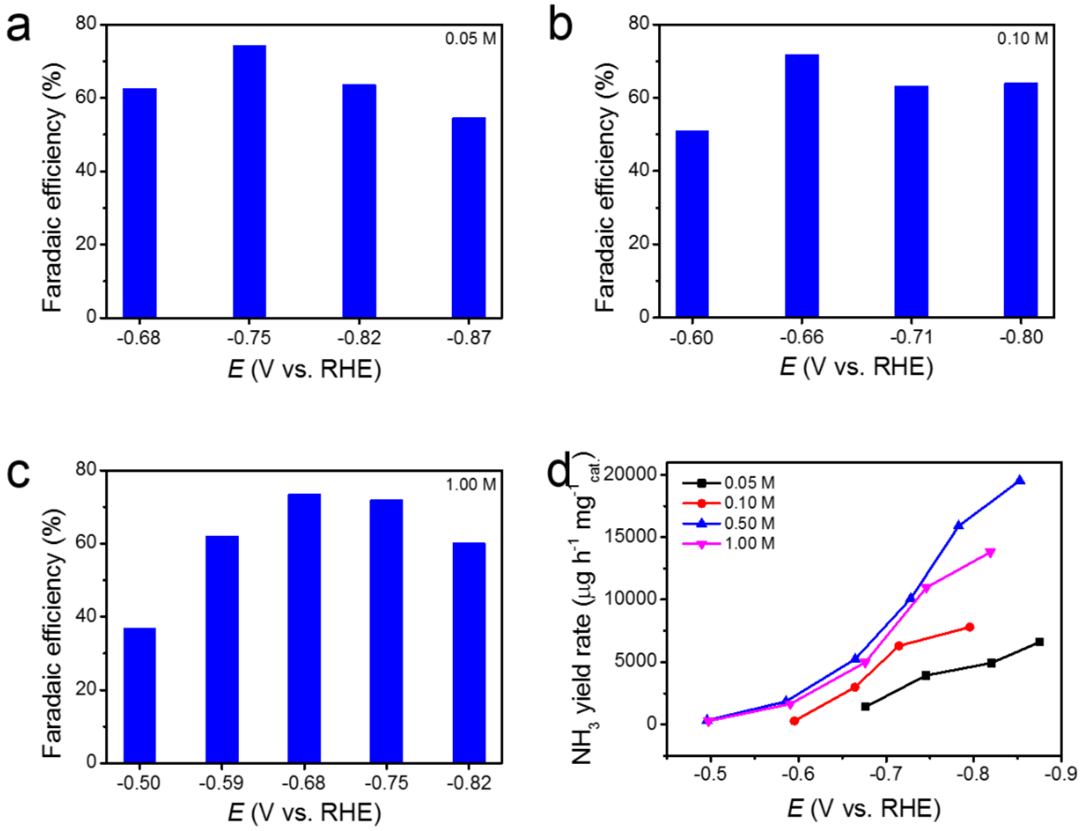

Supplementary Figure 30. NH_3 (a-c) FE and (d) yield rate of NO_3^- reduction on Fe SAC at different concentrations of KNO_3 at each given potential.

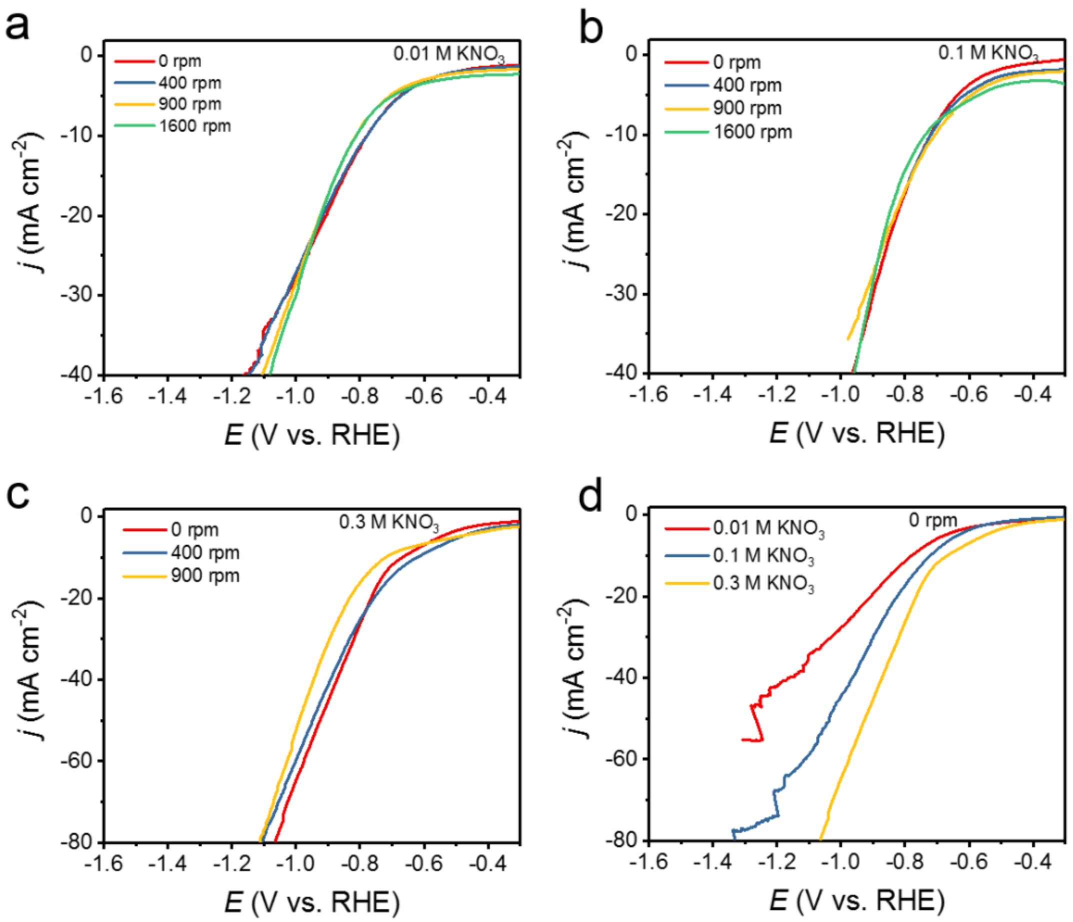

Supplementary Figure 31. LSV curves on rotating disk electrode (RDE). (a-c) LSV curves of the Fe SAC with various rotation rates in different concentrations of KNO_3 electrolyte. (d) LSV curves of the Fe SAC with a rotation rate of 0 rpm in different concentrations of KNO_3 electrolyte. It is found that the LSV curves are very close under different rotation rates in the same concentration of KNO_3 electrolyte including 0.01, 0.1 and 0.3 M KNO_3, suggesting that the activity of Fe SAC has no relationship with rotation rates or the mass diffusion.

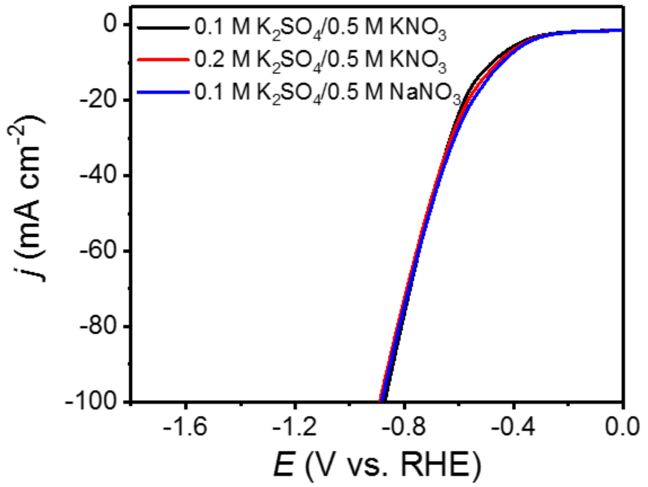

Supplementary Figure 32. LSV curves of the Fe SAC in 0.50 M KNO_3/0.10 M K_2SO_4, 0.50 M KNO_3/0.20 M K_2SO_4 and 0.50 M $NaNO_3$/0.10 M K_2SO_4 electrolytes. The LSV curves were very close, thus the ammonia yield rate is related to the concentration of NO_3^-, not K^+.

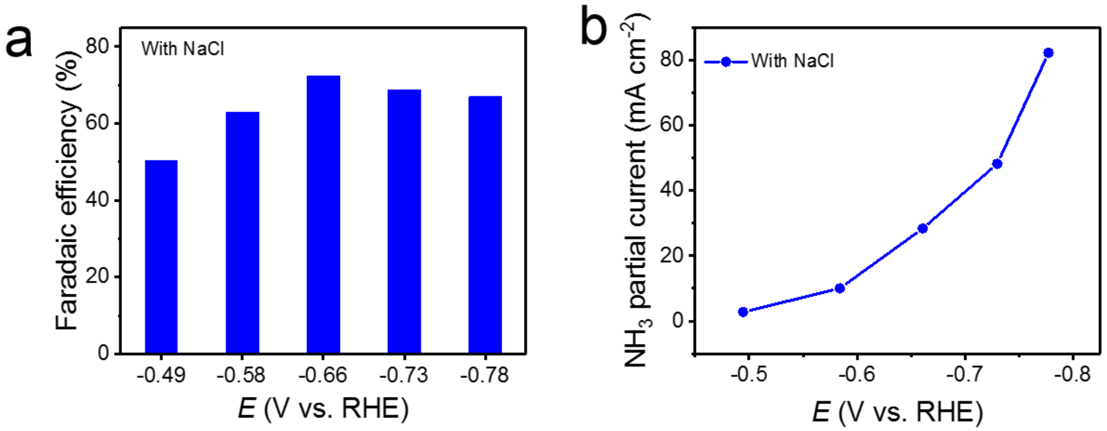

Supplementary Figure 33. (a) NH_3 FE of Fe SAC at each given potential for 0.5 h electrocatalysis test with NaCl in electrolyte on both anode and cathode sides. (b) NH_3 partial current density of Fe SAC at each given potential for 0.5 h electrocatalysis tests with NaCl in electrolyte.

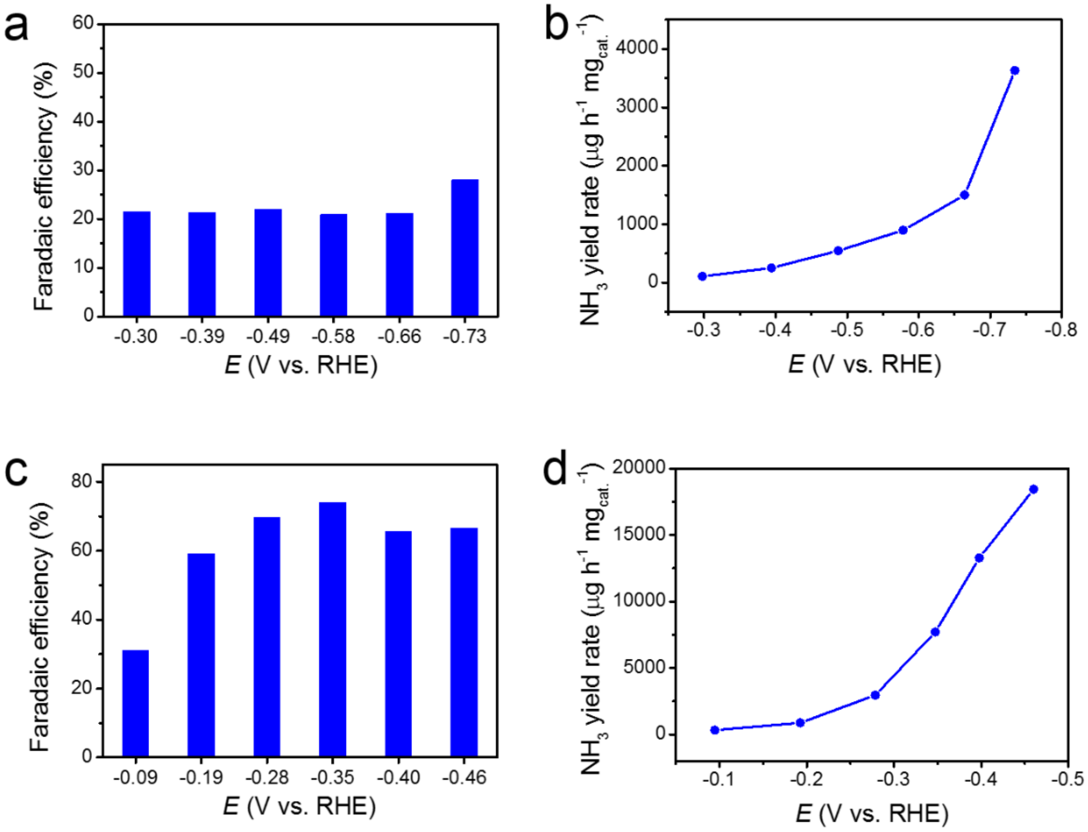

Supplementary Figure 34. NH_3 (a) FE and (b) yield rate of NO_3^- reduction on Fe SAC in acidic KNO_3 solution (pH=1) at each given potential. NH_3 (c) FE and (d) yield rate of NO_3^- reduction on Fe SAC in alkaline KNO_3 solution (pH=13) at each given potential.

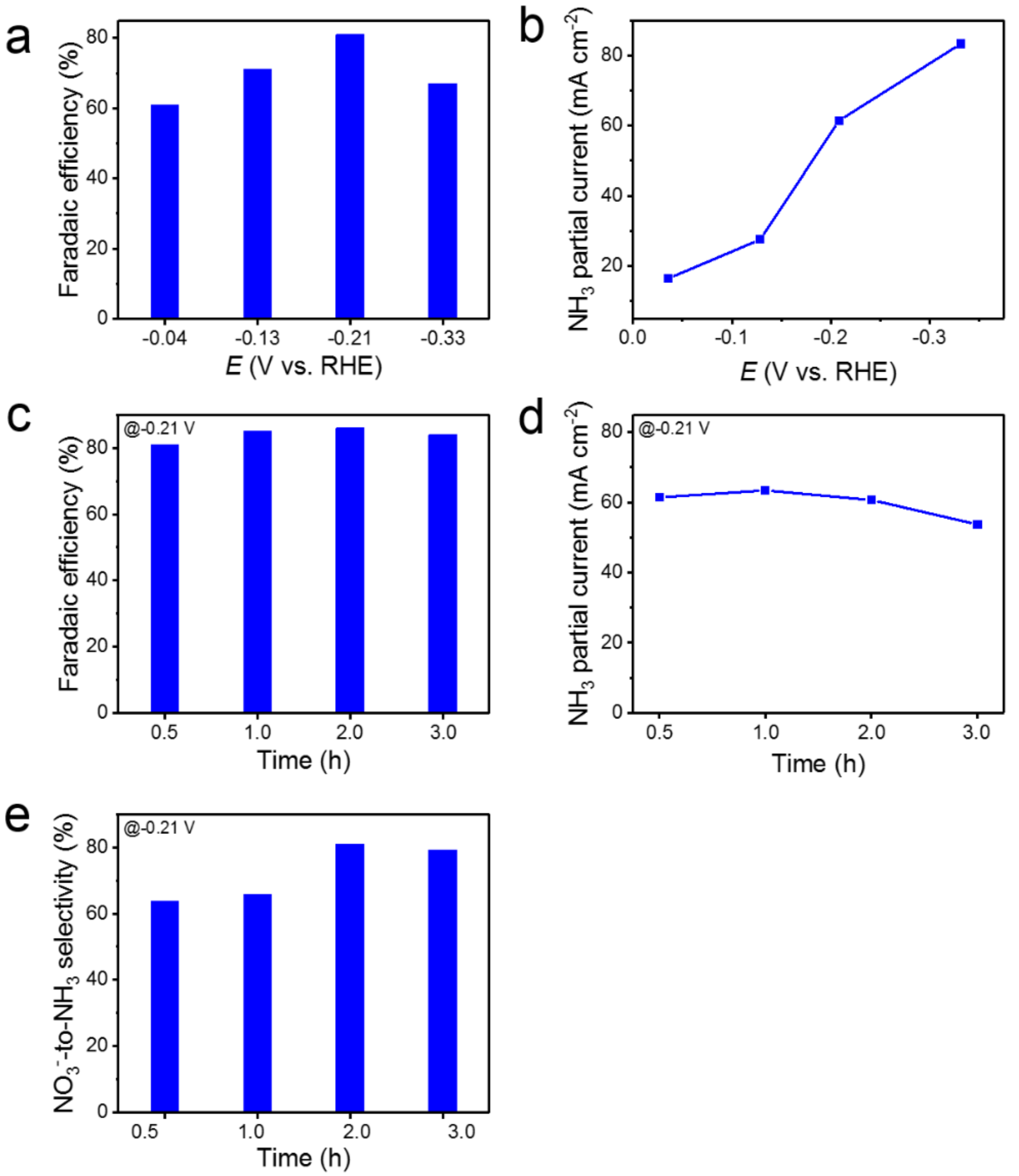

Supplementary Figure 35. Electrocatalytic nitrate reduction performance of Fe SAC loaded on carbon paper tested in 0.1 M KNO_3/1.0 M KOH mixed electrolyte. Catalyst loading content: 1.0 mg cm^{-2}. (a) NH_3 FE and (b) NH_3 partial current density of Fe SAC at each given potential for 0.5 h electrocatalysis test. (c) NH_3 FE, (d) NH_3 partial current density, and (e) NO_3^--to-NH_3 selectivity of Fe SAC at -0.21 V at different time. By increasing the loading of Fe SAC to 1.0 mg cm^{-2} and using 0.1 M KNO_3/1. 0 M KOH mixed solution as the electrolyte, the best FE can be enhanced to 81% at -0.21 V for 0.5 h electrocatalysis test. Furthermore, when we prolonged the electrocatalysis reaction time from 0.5 h to 2 h at -0.21 V, the FE can be up to 86% at 2 h test. The best NO_3^--to-NH_3 selectivity of Fe SAC can be up to 81.0%. The possible reason that prolonging the electrocatalysis reaction time from 0.5 h to 2 h at -0.21 V can enhance FE is as follows.

With the increase of electrocatalysis test time, the concentration of byproduct NO_2^- would increase in the electrolyte. As NO_2^- reduction to NH_3 was much easier than NO_3^- reduction (Supplementary Figure 29), thus the FE of NO_3^--to-NH_3 would increase from 0.5 h to 2 h. Further prolongation of electrocatalysis test time would cause the decrease the concentration of NO_3^-or NO_2^-, thus HER could happen and the FE of NO_3^--to-NH_3 would decrease. Indeed, we found that the FE of NO_3^--to-NH_3 decreased from 86% at 2-h to 84% at 3-h test, and the NH_3 partial current density also decreased accordingly.

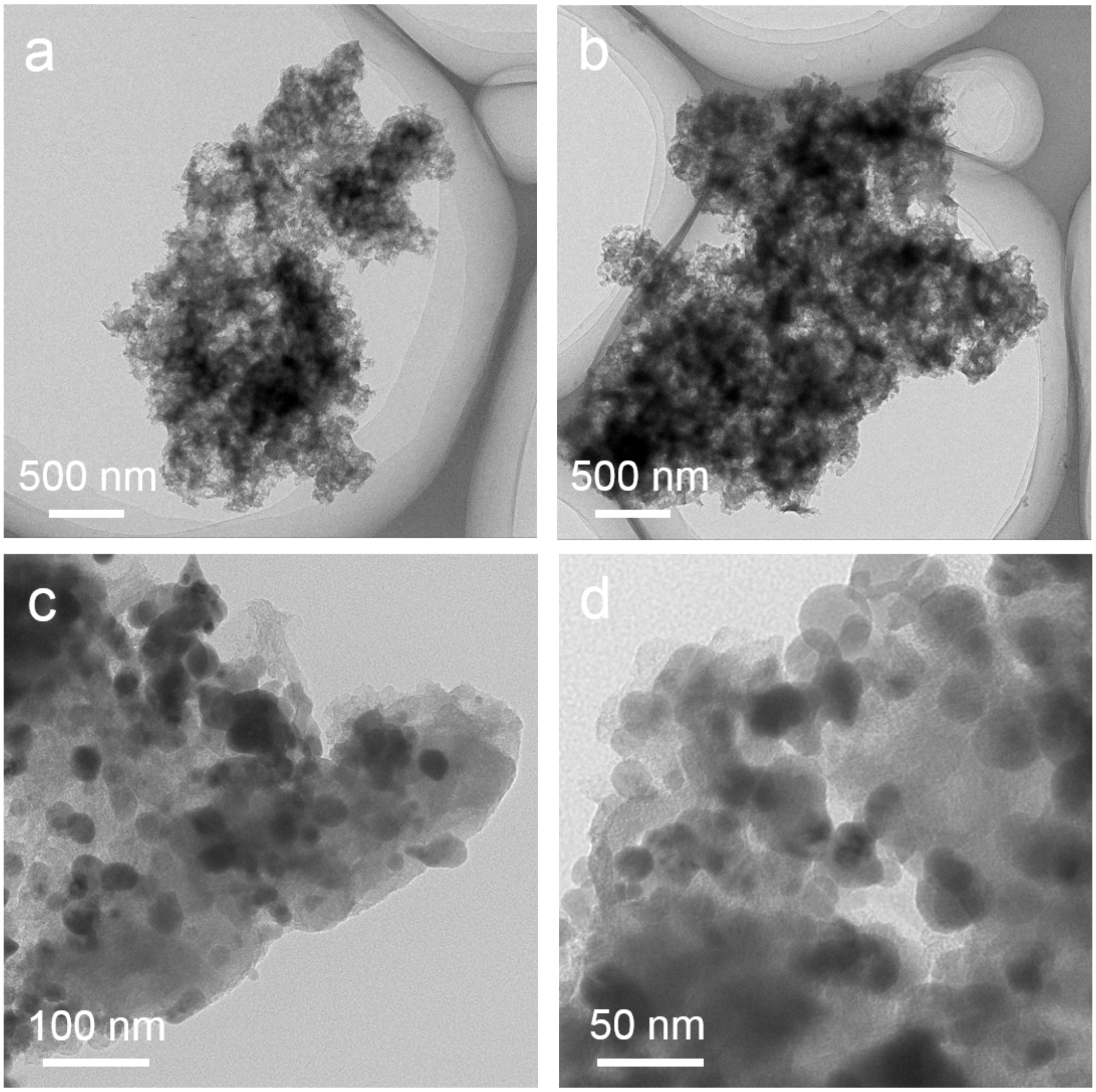

Supplementary Figure 36. TEM images of (a, b) NC and (c, d) FeNP/NC. The NC shows a mesoporous strcture, similar to that of Fe SAC. Some nanoparticles on carbon supports are observed for FeNP/NC sample.

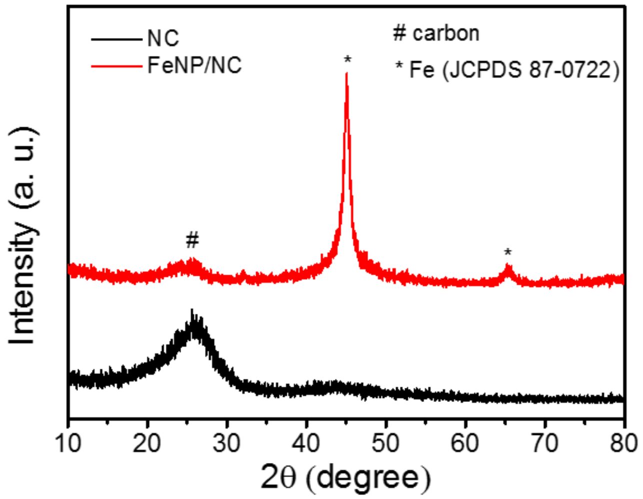

Supplementary Figure 37. XRD patterns of NC and FeNP/NC. The XRD result of FeNP/NC confirms that the nanoparticles in FeNP/NC are metallic Fe (JCPDS 87-0722).

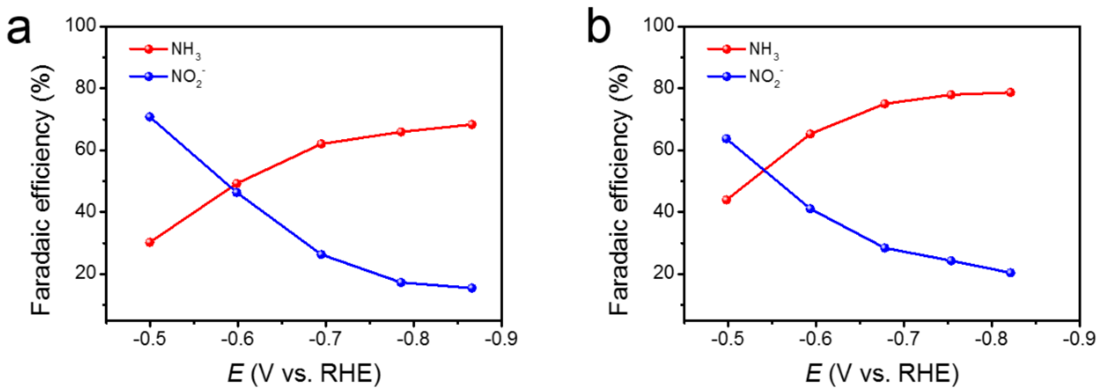

Supplementary Figure 38. FE of NO_3^- reduction on (a) NC and (b) FeNP/NC catalysts at each given potential.

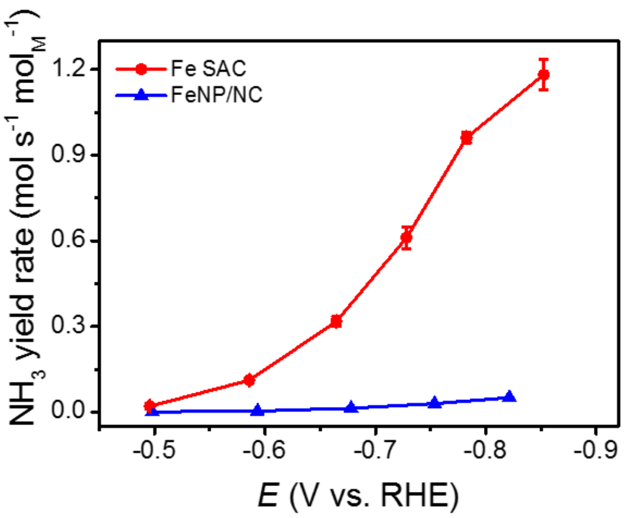

Supplementary Figure 39. NH_3 yield rate of Fe SAC, and FeNP/NC based on the metal content.

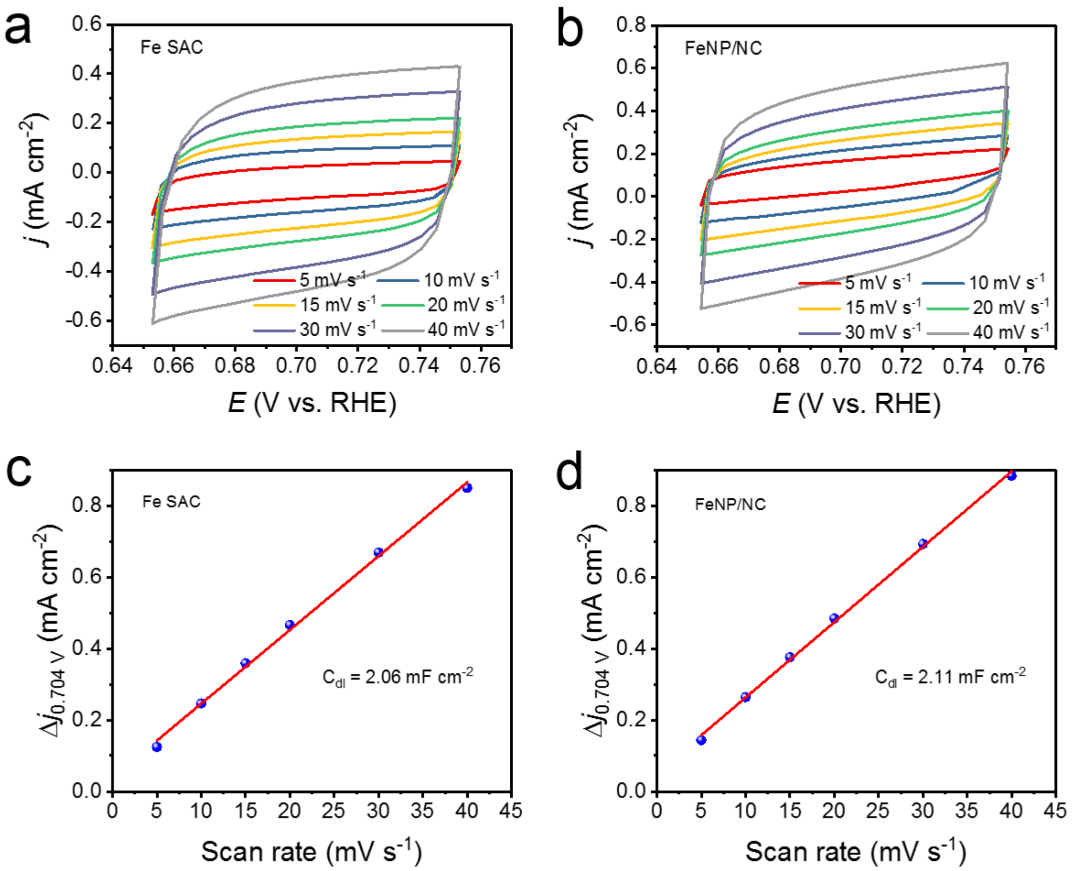

Supplementary Figure 40. Cyclic voltammograms (CV) for (a) Fe SAC and (b) FeNP/NC catalysts at different scan rates from 5 to 40 mV s^{-1}, respectively. Plots showing the extraction of the C_{dl} for (c) Fe SAC and (d) FeNP/NC, respectively. The double-layer capacitance (C_{dl}) is proportional to the electrochemical surface area (ECSA).

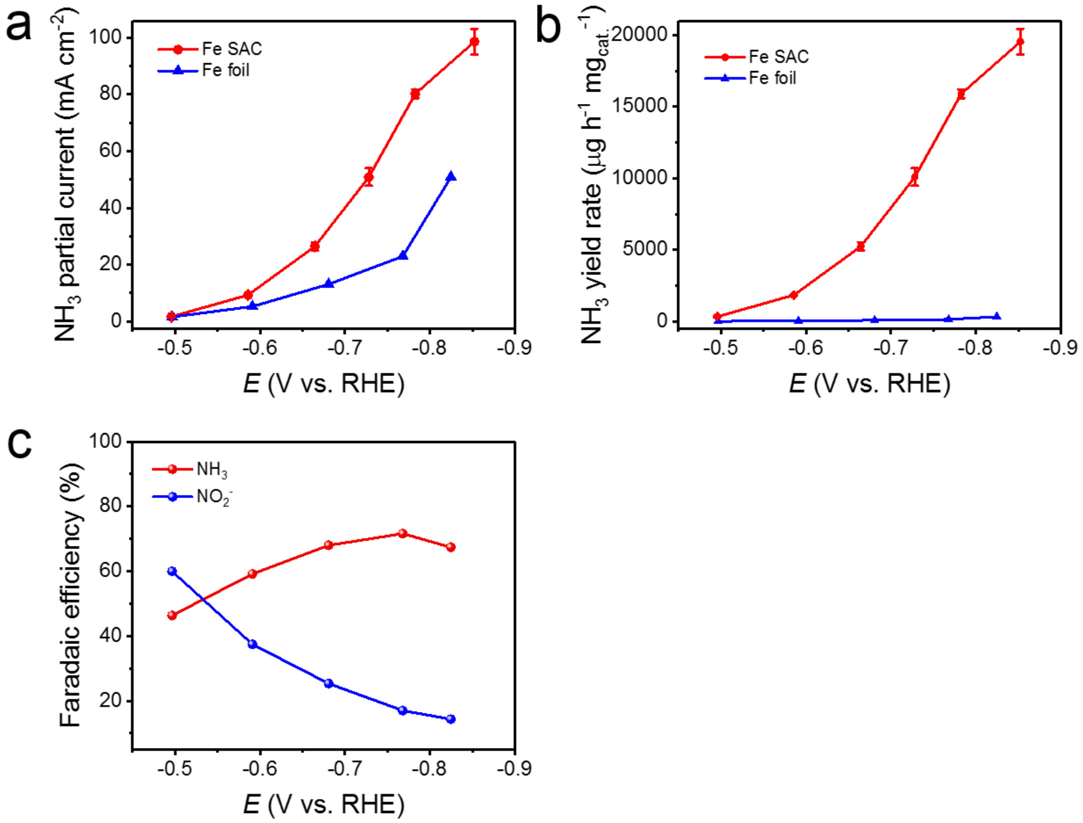

Supplementary Figure 41. (a) NH_3 partial current density and (b) NH_3 yield rate based on the catalyst loading amount of Fe SAC and Fe foil. (c) FE of NO_3^- reduction on Fe foil.

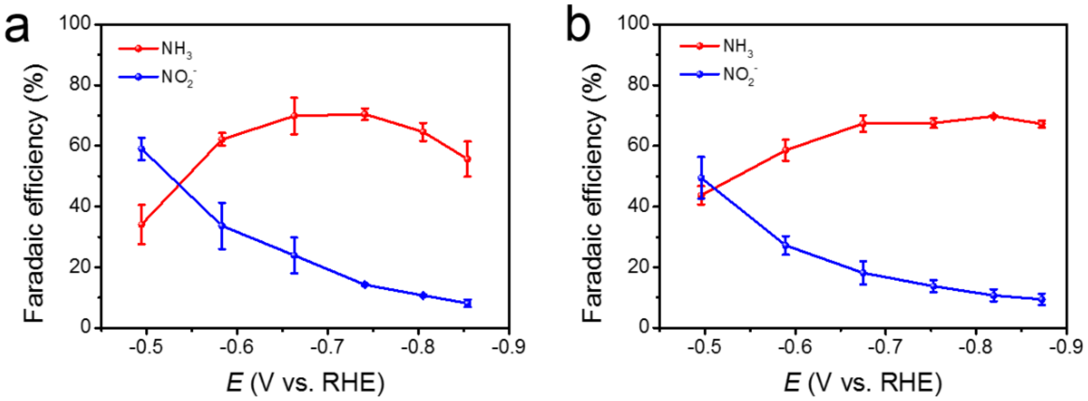

Supplementary Figure 42. FE of NO_3^- reduction on (a) Co SAC and (b) Ni SAC catalysts at each given potential.

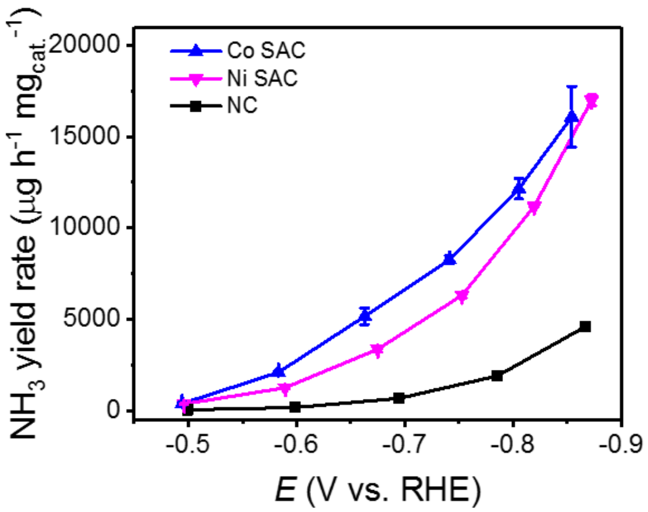

Supplementary Figure 43. NH_3 yield rate of Co SAC, Ni SAC and NC.

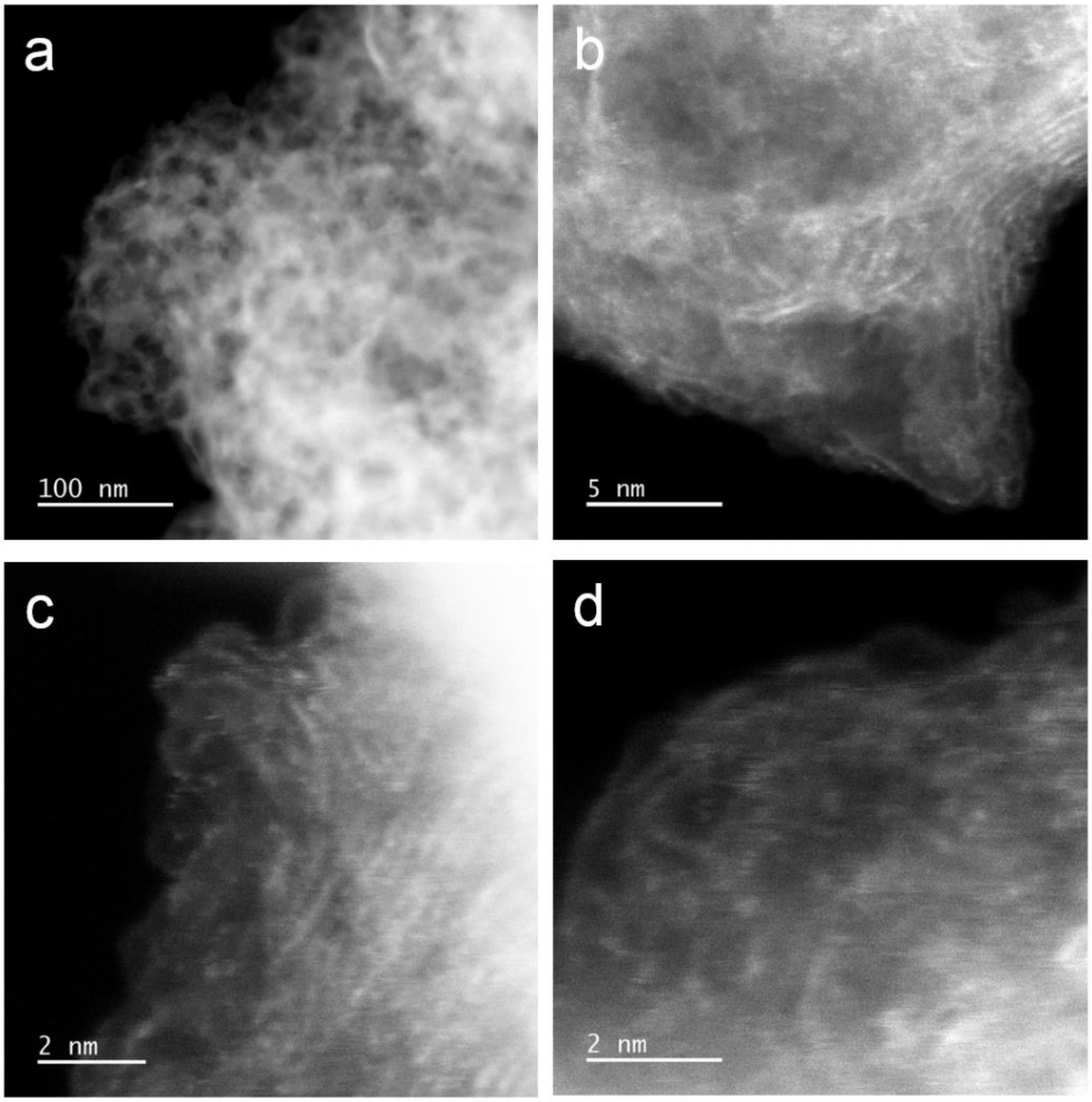

Supplementary Figure 44. (a) MAADF-STEM and (b-d) AC MAADF-STEM images of Fe SAC after 20 electrolysis cycles. We only find individual metal atoms in recycled Fe SAC. No cluster or nanoparticle is observed.

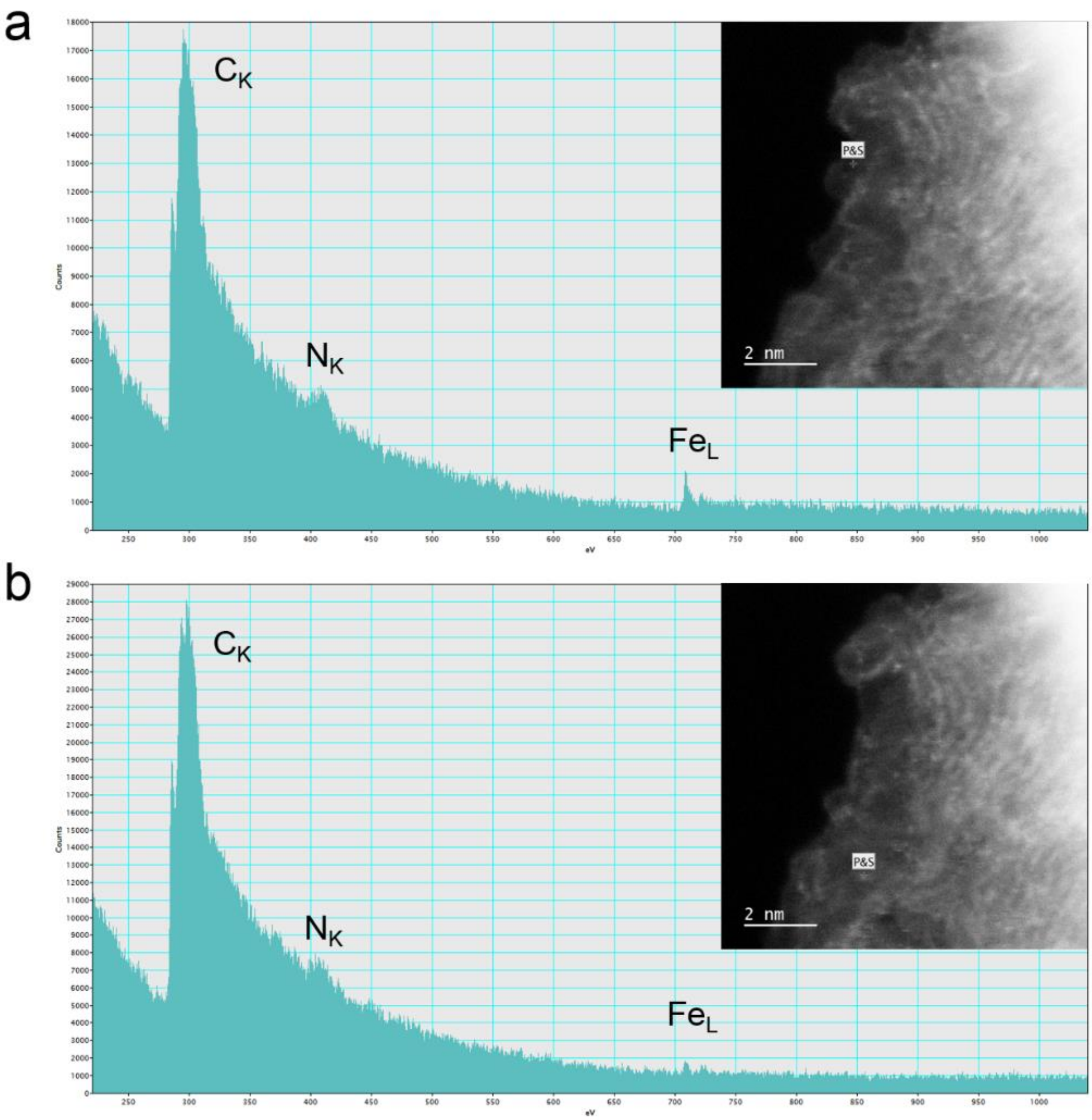

Supplementary Figure 45. (a, b) EELS point spectra from the marked areas in the inserted AC MAADF-STEM images of Fe SAC after 20 electrolysis cycles. Insets are AC MAADF-STEM images of Fe SAC after 20 electrolysis cycles at different areas. Fe-N-C coordinate structures are still existed in these areas.

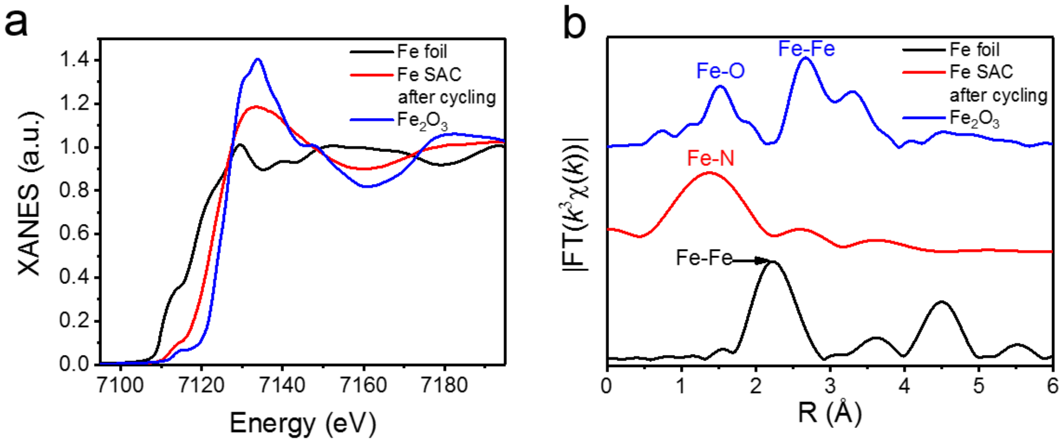

Supplementary Figure 46. (a) XANES spectra at the Fe K-edge of the Fe SAC after cycling, referenced Fe foil and Fe_2O_3. (b) FT k^3-weighted $\chi(k)$-function of the EXAFS spectra at Fe K-edge.

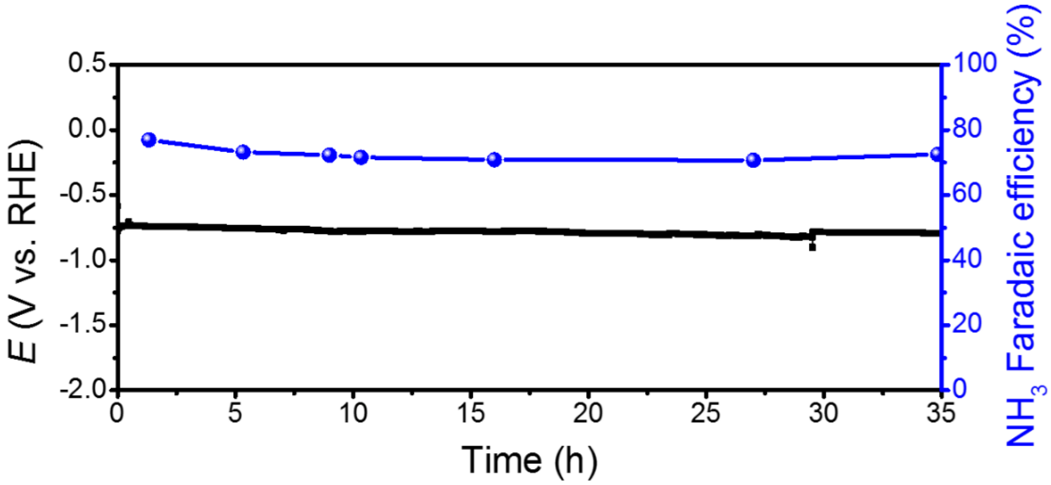

Supplementary Figure 47. Stability test of the Fe SAC for NO_3^- reduction using a flow cell at 35 mA cm^{-2}.

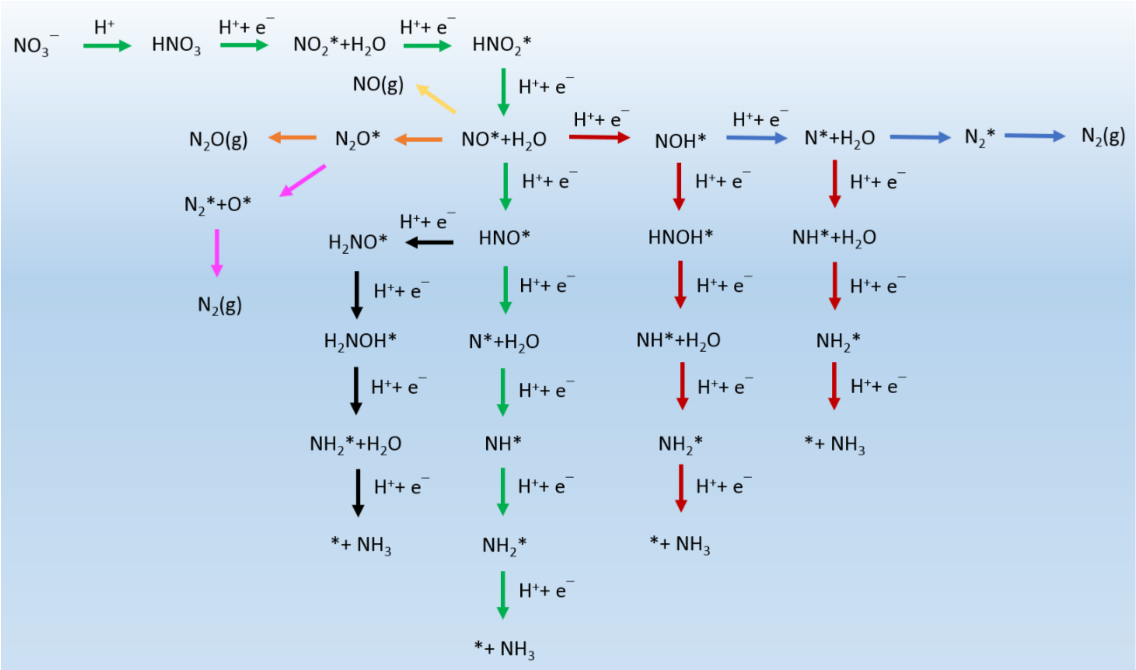

Supplementary Figure 48. Full reaction network for NO_3^- reduction reaction. To avoid difficulties in accurately describing the electronic structure of NO_3^-, we calculated the adsorption energies of each intermediate with reference to HNO_3 as suggested by Calle-Vallejo et al[1]. In this scheme, the binding energy of each intermediate listed in Supplementary Fig. 48 is calculated with respect to hydrogen and nitric acid in the gas phase. Below is the list of the reactions used for calculation of each step.

$HNO_3 + 5/2H_2 + * \rightarrow N* + 3H_2O$

$HNO_3 + 3H_2 + * \rightarrow NH* + 3H_2O$

$HNO_3 + 7/2H_2 + * \rightarrow NH_2* + 3H_2O$

$HNO_3 + 4H_2 + * \rightarrow NH_3* + 3H_2O$

$HNO_3 + 2H_2 + * \rightarrow NOH* + 2H_2O$

$HNO_3 + 2H_2 + * \rightarrow HNO* + 2H_2O$

$HNO_3 + 5/2H_2 + * \rightarrow H_2NO* + 2H_2O$

$HNO_3 + 5/2H_2 + * \rightarrow HNOH* + 2H_2O$

$HNO_3 + 3H_2 + * \rightarrow H_2NOH* + 2H_2O$

$HNO_3 + 1/2H_2 + * \rightarrow NO_2* + H_2O$

$NO + 2H_2O + * \rightarrow *NO_3 + 2H_2$

$HNO_3 + 3/2H_2 + * \rightarrow NO* + 2H_2O$

$HNO_3 + * \rightarrow HNO_3*$

$H_2O + * \rightarrow OH* + 1/2H_2$

$H_2O + * \rightarrow O* + H_2$

$HNO_3 + H_2 + * \rightarrow HNO_2* + H_2O$

$HNO_3 + H_2 + * \rightarrow NO_2H* + H_2O$

$HNO_3 + 3/2H_2 \rightarrow NO(g) + 2H_2O$

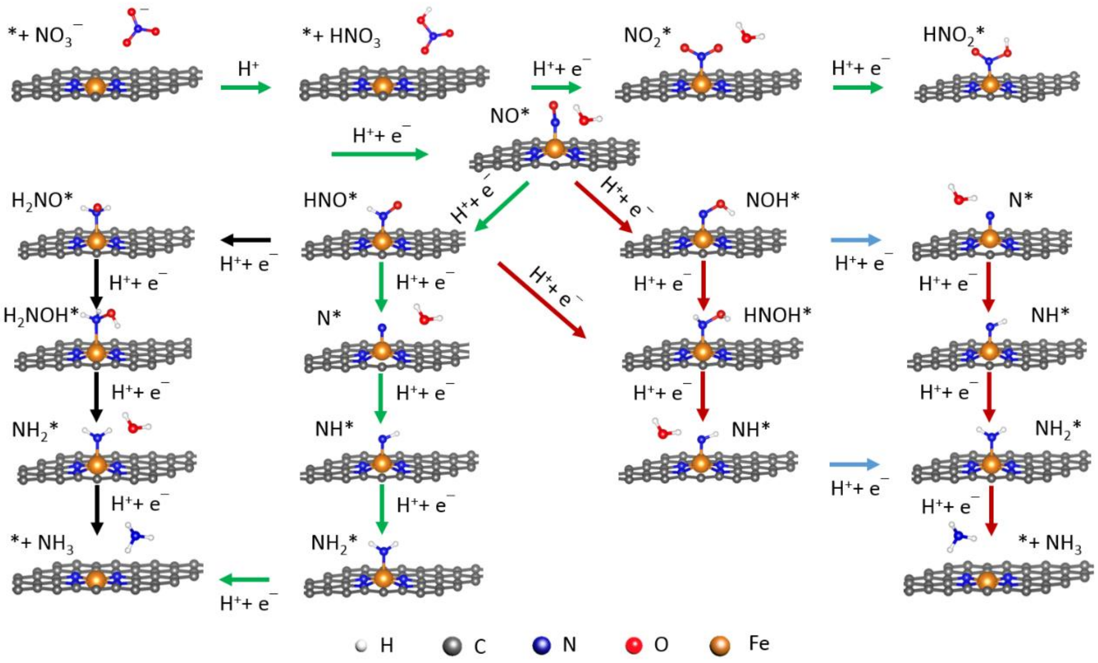

Supplementary Figure 49. All the reaction pathways that result in NH_3 as the main product. The minimum energy pathway is shown by green arrows.

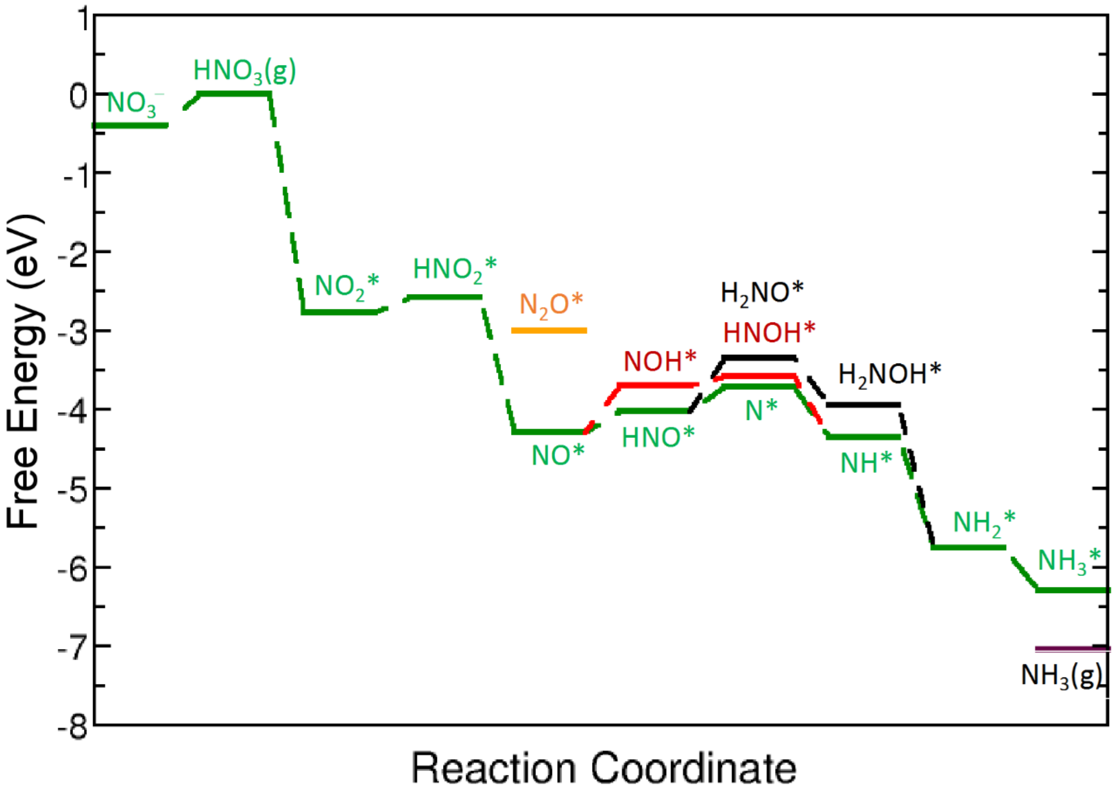

Supplementary Figure 50. Calculated free energy diagram of all different possible intermediates and their energy profile across reaction coordinate that result in NH_3 product on Fe SAC. The minimum energy pathway is shown by green. Fig. 4a (also the green arrows in Supplementary Fig. 49) and green line in Fig. 4b indicate the minimum energy pathway (MEP) for NO_3^- reduction to NH_3 on the Fe SAC.

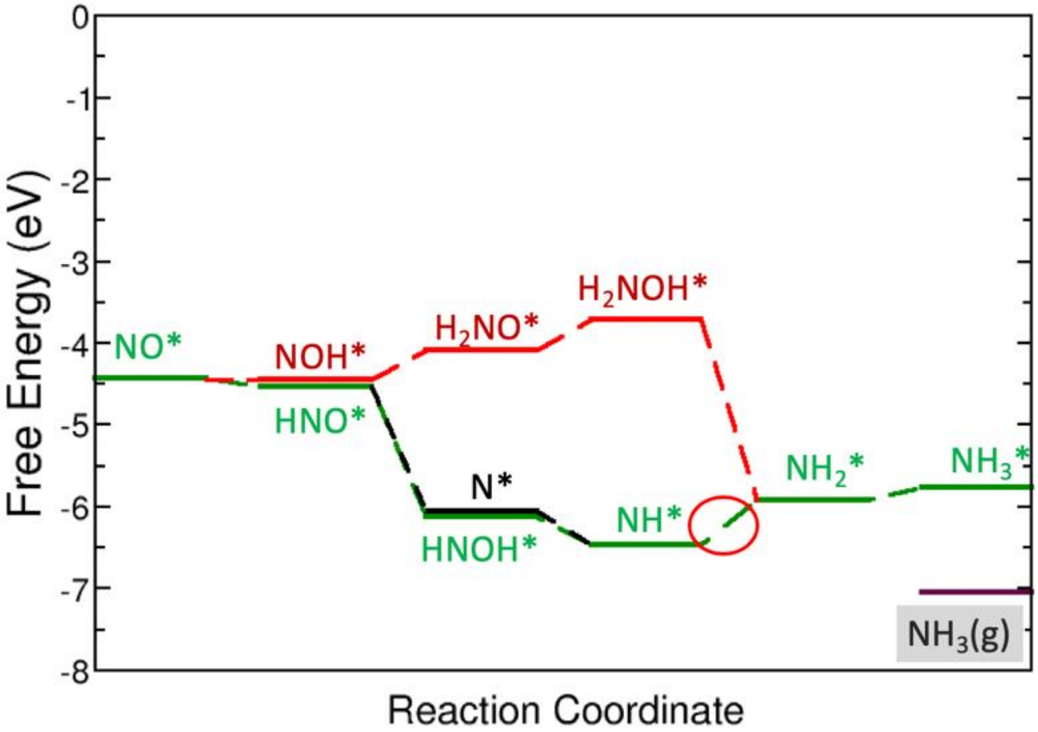

Supplementary Figure 51. Free energy diagram for different possible pathways on Fe(110). The minimum energy pathway is shown in green. Red circle shows the thermodynamic barrier. Of note, since reduction of NO_3^- to NO* is thermodynamically facile, we only focus on the free energy diagram of NO* reduction to NH_3.

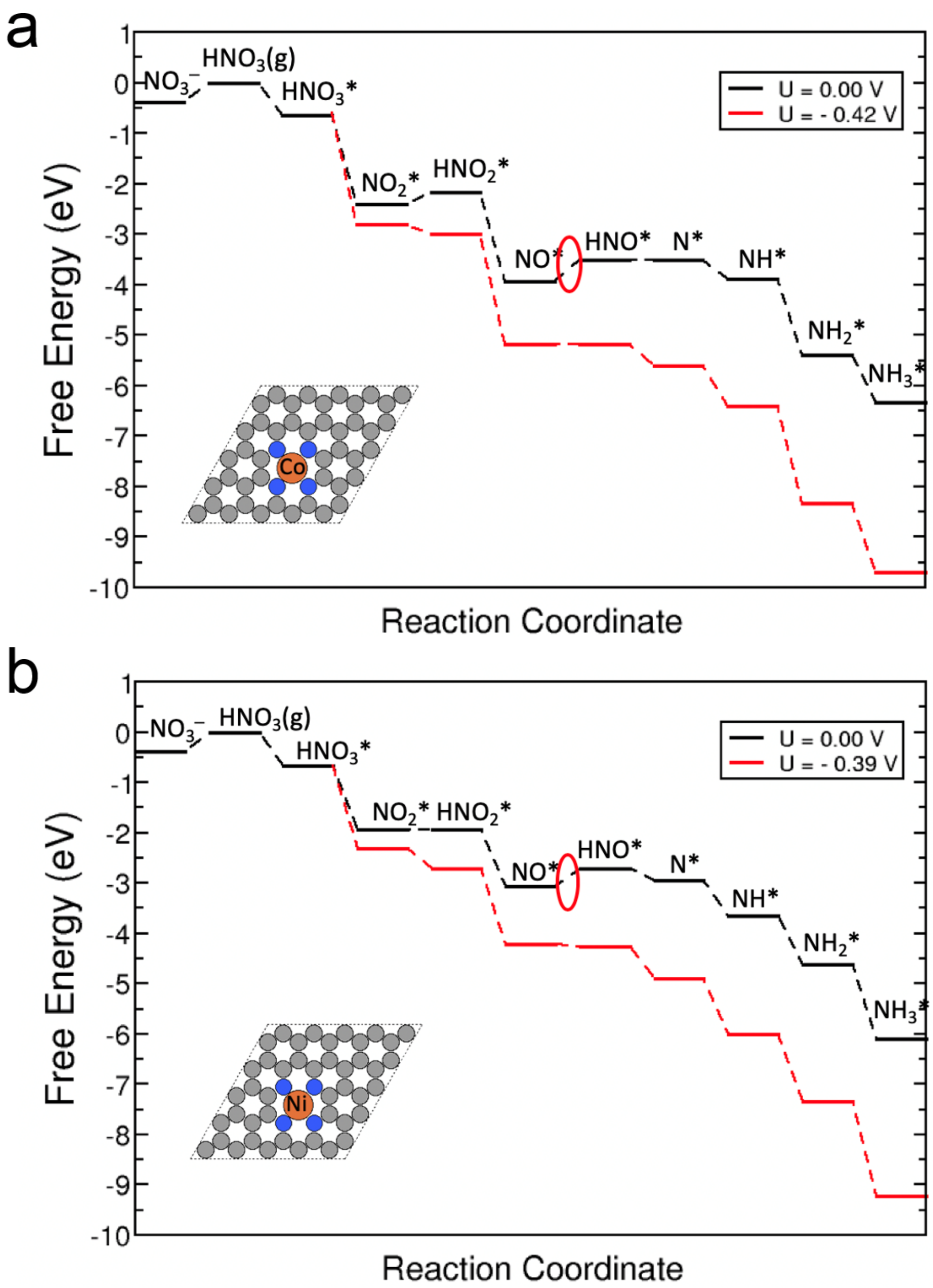

Supplementary Figure 52. Free energy diagrams for (a) Co and (b) Ni SACs. The thermodynamic barriers are shown with red ovals. Black and red pathways are the free energy diagrams at zero potential and the working potential where all steps become downhill in free energy, respectively.

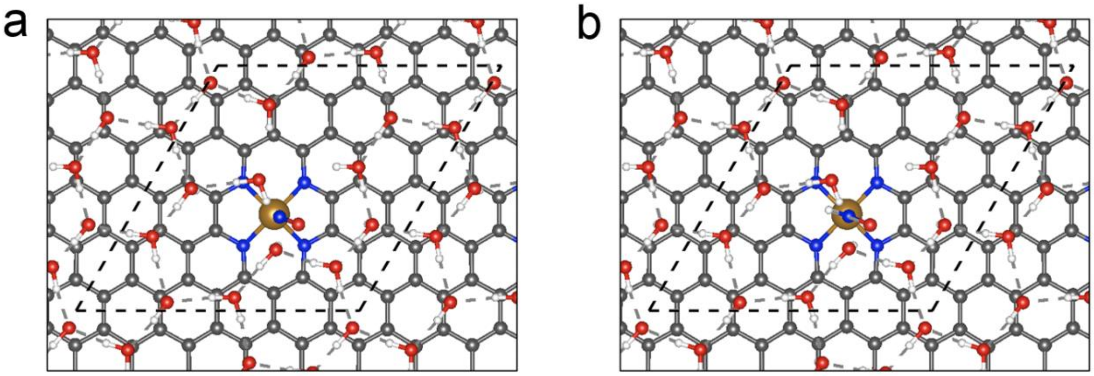

Electrochemical Step	$\Delta G_{no\ solvent}$ (eV)	$\Delta G_{with\ solvent}$ (eV)	$U_{L,\ no\ solvent}$ (V)	$U_{L,\ with\ solvent}$ (V)
NO*→HNO*	0.278	0.277	0.278	0.277

Supplementary Figure 53. Solvation Effect. The optimized structures of (a) NO* and (b) HNO* intermediates by inclusion of explicit solvation models. To examine the solvation effect on the adsorption energies of the intermediates we used an explicit solvation model, which is optimized for our graphene model structure and agrees well with the literature[2]. This analysis indicates a negligible shift (0.001 V) in the calculated limiting potential by inclusion of the solvation. This small effect won't change the conclusion of this study. The experiment trend can be well captured without including a solvation effect.

Supplementary Table 1. Textural properties of Fe SAC, Co SAC and Ni SAC.

Sample	BET surface area ($m^2 g^{-1}$)	Total pore volume ($cm^3 g^{-1}$)	Average pore diameter (nm)
Fe SAC	285.8	0.80	11.1
Co SAC	425.7	0.92	8.7
Ni SAC	460.8	1.17	10.1

Supplementary Table 2. Structural parameters of Fe SAC, Co SAC, Ni SAC, Fe foil and Fe_2O_3 extracted from the EXAFS fitting.

Sample	Shell	N	R (Å)	σ^2 (10^{-3}Å^2)	ΔE_0 (eV)	R factor
Fe foil	Fe-Fe 1st shell	8(fixed)	2.419±0.010	2.9±0.5	1.434±1.044	0.003
	Fe-Fe 2nd shell	6(fixed)	2.843±0.011	3.0±0.9		
Fe SAC	Fe-N	4.40±0.10	1.922±0.008	0.2±0.6	8.65±0.69	0.008
Fe_2O_3	Fe-O 1st shell	3(fixed)	1.896±0.007	2.8±1.2	9.99±0.30	0.017
	Fe-Fe 2nd shell	3(fixed)	2.836±0.013	3.3±0.7	3.22±2.06	
Co SAC	Co-N	4.36±0.38	1.947±0.010	11.5±1.7	7.63±0.90	0.014
Ni SAC	Ni-N	4.45±0.30	1.923±0.007	4.0±0.9	7.93±1.63	0.027

N is the coordination number; R is interatomic distance (the bond length between central atoms and surrounding coordination atoms); σ^2 is Debye-Waller factor (a measure of thermal and static disorder in absorber-scatterer distances); ΔE_0 is edge-energy shift (the difference between the zero kinetic energy value of the sample and that of the theoretical model). R factor is used to value the goodness of the fitting.

Supplementary Table 3. Comparison of performance of Fe SAC with reported catalysts by electrocatalytic nitrate reduction. Of note, it is might be unfair to compare the intrinsic nitrate reduction activity and selectivity of Fe SAC with most of the reported materials, because the past nitrate reduction mainly focus on nitrate removal with N_2 as the target product.

Catalyst	Electrolyte	NH_3 partial current	FE (%)	NO_3^--to-NH_3 selectivity (%)[a]	Reference
**Fe SAC**	**0.5 M KNO_3/0.1 M K_2SO_4**	**98.6 mA cm^{-2} 16324.5 A g_{Fe}^{-1} (-0.85 V)**	**74.9 (-0.66V)**	**~69 (-0.85 V)**	**This work**
	**0.1 M KNO_3/1.0 M KOH**	**60.7 mA cm^{-2} 4019.9 A g_{Fe}^{-1} (-0.21 V, 2 h)**	**86 (-0.21 V, 2 h)**	**81 (-0.21 V, 2 h)**	
Sn	0.05 M KNO_3 + 0.1 M K_2SO_4	/	/	8	3
CL-Fe@C	100 mg L^{-1} NO_3^- -N + 0.02 M NaCl	/	/	<2	4
Cu	0.1 M $NaNO_3$ + 0.01 M NaOH + 0.5 M NaCl	/	/	26.4	5
Co_3O_4-TiO_2/Ti	50 ppm NO_3^- + 0.1 M Na_2SO_4 + PVP + 1000 ppm Cl	/	/	24	6
Pd_4Cu_4@N-pC	neutral NO_3^- solution	/	/	<20	7
Cu/rGO/graphite plate (GP)	0.02 M $NaNO_3$ + 0.02 M NaCl	/	/	29.9	8
Ni-Fe^0@ Fe_3O_4	50 ppm NO_3^- + 10 mM NaCl	/	/	10.4	9
Pd-Cu/SS	0.01 M $NaClO_4$ + 0.6 mM $NaNO_3$	/	/	6	10
TiO_{2-x}/Ti foil	0.5 M Na_2SO_4 + 50 ppm NO_3^- -N	<10 mA cm^{-2} <20 A g_{Ti}^{-1}	85	87.1	11
Pd-Cu/γAl_2O_3	50 ppm NO_3^- -N	/	/	19.6	12
Cu/Cu_2O NWAs	200 ppm nitrate-N + 0.5 M Na_2SO_4	~100 mA cm^{-2} ~200 A g_{Cu}^{-1}	95.8	81.2	13

[a] NO_3^--to-NH_3 selectivity = $C_{NH3}/\Delta C_{NO3-}$ × 100%, where C_{NH3} is the concentration of NH_3(aq), and ΔC_{NO3-} is the concentration difference of NO_3^- before and after electrolysis. In main text, the selectivity mentioned refers to FE unless otherwise specified. Since Fe single atoms are active sites for Fe SAC, we also compared the metal mass activity of different catalysts. For TiO_{2-x} and Cu/Cu_2O NWAs on metal foils, the loading of catalysts is always high. We assumed a very low metal loading of 0.5 mg_{metal} cm^{-2} for these catalysts on their surface, based on which we estimated NH_3 partial current per mass of metal content.

Supplementary Note 1. Synthesis and characterizations of Co and Ni single atoms on nitrogen-doped mesoporous carbon (Co SAC and Ni SAC).

Co SAC and Ni SAC, were also prepared for comparison in this study. TEM observations and N_2 sorption analysis reveals the highly mesoporous structures of Co SAC and Ni SAC (Supplementary Figs. 8, 9). Their BET surface area and pore volume are summarized in Supplementary Table 1. HRETM, XRD and Raman spectra indicate the graphitic carbon structures of Co SAC and Ni SAC (Supplementary Figs.10-12). AC MAADF-STEM images, and EDS mapping analysis, XRD, FT k^3-weighted EXAFS profiles, and WT of K-edge EXAFS oscillations show the Co, Ni are atomically dispersed on N-doped carbon (Supplementary Figs.11, 13-18). The content of Co and Ni determined by ICP-OES analysis are 4.47 and 5.28 wt%, respectively. EELS point spectra, XPS and EXAFS spectra at K-edge show Co, Ni single atoms are coordinated with N in Co SAC and Ni SAC (Supplementary Figs. 17, 19, 20). The coordination numbers of Co in Co SAC and Ni in Ni SAC are also about 4 (Supplementary Fig. 21, and Supplementary Table 2). All of these characterizations clearly indicate that Co SAC and Ni SAC with very similar structure to the Fe SAC have been successfully prepared, and the TM-assisted carbonization method is a general strategy to synthesize SACs.

Supplementary Note 2. The comparison of the catalytic performance over Fe SAC and Fe NP catalyst.

First, we noticed that during the synthesis of Fe NPs on NC, it was inevitable that there were Fe single atomic sites existing, which could also contribute to the catalytic activity as we observed from the Fe SAC sample. Second, it is difficult for us to figure out what are the well-defined active sites in Fe NP sample. It could be one certain Fe crystal facets, a neighboring pair of Fe atoms, or the interface between FeNP and NC. Due to the complicity of nitrate reaction pathways and the surface structure of Fe NPs, it is difficult for us to clearly identify the active sites of the FeNP/NC catalyst by DFT calculations. Third, we noticed that the Fe NP catalyst is not stable during nitrate reduction tests: we found that ~ 20% of the Fe content was dissolved into the electrolyte solution at -0.87 V after 0.5-h. The chemical reaction between nitrate and Fe also resulted in a part of the produced ammonia, and it is difficult quantify the Fe active sites for electrocatalytic nitrate reduction. According to the above three points, it is difficult to calculate the intrinsic TOF of Fe NP catalysts based on its surface atoms. What we want to emphasize in this comparison is to simply demonstrate that Fe single atoms are much more active than that of Fe NPs. Therefore, to avoid any misinterpretation, we directly compared the ammonia yield rate between Fe SAC and Fe NP catalysts based on the catalyst loading amount (Figure 3c), and ammonia yield rate based on the mass of Fe metal (Supplementary Fig. 39).

Supplementary Note 3. Comparison of different SACs including Fe, Co and Ni SACs for nitrate reduction.

Due to very similar performance to Fe SAC in various electrocatalytic reactions[14], Co SAC and Ni SAC were also tested for comparison in this study (Fig. 3f). The metal content normalized NH_3 yield rate of the Co SAC catalyst are 0.111±0.0096 mol s^{-1} mol_M^{-1} at -0.66 V, 0.346 ± 0.0358 mol s^{-1} mol_M^{-1} at -0.85V, respectively, which are only 35.0% and 29.3% of those of the Fe SAC catalysts at the same potentials. The Ni SAC shows the lowest NH_3 yield rate under studied potential range. Thus, the activities of these SACs follow the trend of Fe SAC > Co SAC > Ni SAC. Additionally, Co SAC and Ni SAC need more negative potentials to get the best FEs, i.e. 70.4% at -0.74 V for Co SAC, and 69.8% at -0.82 V for Ni SAC (Supplementary Fig. 42), further indicating that Fe SAC possesses better nitrate reduction performance than Co SAC and Ni SAC[15].

Supplementary references

1. Calle-Vallejo, F., Huang, M., Henry, J.B., Koper, M.T.M. & Bandarenka, A.S. Theoretical design and experimental implementation of Ag/Au electrodes for the electrochemical reduction of nitrate. *Phys. Chem. Chem. Phys.* **15**, 3196-3202 (2013).
2. Kirk, C. *et al.* Theoretical Investigations of the Electrochemical Reduction of CO on Single Metal Atoms Embedded in Graphene. *ACS Cent. Sci.* **3**, 1286-1293 (2017).
3. Katsounaros, I., Ipsakis, D., Polatides, C. & Kyriacou, G. Efficient electrochemical reduction of nitrate to nitrogen on tin cathode at very high cathodic potentials. *Electrochim. Acta* **52**, 1329-1338 (2006).
4. Su, L. *et al.* Tailoring the Assembly of Iron Nanoparticles in Carbon Microspheres toward High-Performance Electrocatalytic Denitrification. *Nano Lett.* **19**, 5423-5430 (2019).
5. Reyter, D., Bélanger, D. & Roué, L. Nitrate removal by a paired electrolysis on copper and Ti/IrO_2 coupled electrodes – Influence of the anode/cathode surface area ratio. *Water Res.* **44**, 1918-1926 (2010).
6. Gao, J. *et al.* Non-precious Co_3O_4-TiO_2/Ti cathode based electrocatalytic nitrate reduction: Preparation, performance and mechanism. *Appl. Catal., B* **254**, 391-402 (2019).
7. Chen, M. *et al.* Achieving high-performance nitrate electrocatalysis with PdCu nanoparticles confined in nitrogen-doped carbon coralline. *Nanoscale* **10**, 19023-19030 (2018).
8. Yin, D. *et al.* In situ growth of copper/reduced graphene oxide on graphite surfaces for the electrocatalytic reduction of nitrate. *Electrochim. Acta* **324**, 134846 (2019).
9. Jonoush, Z.A., Rezaee, A. & Ghaffarinejad, A. Electrocatalytic nitrate reduction using Fe0/Fe_3O_4 nanoparticles immobilized on nickel foam: Selectivity and energy consumption studies. *J. Clean. Prod.* **242**, 118569 (2020).
10. Su, J.F., Ruzybayev, I., Shah, I. & Huang, C.P. The electrochemical reduction of nitrate over micro-architectured metal electrodes with stainless steel scaffold. *Appl. Catal., B* **180**, 199-209 (2016).
11. Jia, R. *et al.* Boosting Selective Nitrate Electroreduction to Ammonium by Constructing Oxygen Vacancies in TiO_2. *ACS Catal.*, 3533-3540 (2020).
12. Zhang, Z. *et al.* Electrochemical-catalytic reduction of nitrate over Pd–Cu/γAl_2O_3 catalyst in cathode chamber: Enhanced removal efficiency and N_2 selectivity. *Chem. Eng. J.* **290**, 201-208 (2016).
13. Wang, Y., Zhou, W., Jia, R., Yu, Y. & Zhang, B. Unveiling the Activity Origin of a Copper-based Electrocatalyst for Selective Nitrate Reduction to Ammonia. *Angew. Chem. Int. Ed.* **59**, 5350-5354 (2020).
14. Fei, H. *et al.* Single atom electrocatalysts supported on graphene or graphene-like carbons. *Chem. Soc. Rev.* **48**, 5207-5241 (2019).
15. Wang, Y. *et al.* Enhanced Nitrate-to-Ammonia Activity on Copper–Nickel Alloys via Tuning of Intermediate Adsorption. *J. Am. Chem. Soc.* **142**, 5702-5708 (2020).
